# Axonal autophagosome maturation defect through failure of ATG9A sorting underpins pathology in AP-4 deficiency syndrome

**DOI:** 10.1080/15548627.2019.1615302

**Published:** 2019-05-29

**Authors:** Davor Ivankovic, James Drew, Flavie Lesept, Ian J. White, Guillermo López Doménech, Sharon A. Tooze, Josef T. Kittler

**Affiliations:** a Neuroscience, Physiology and Pharmacology, UCL, London, UK; b MRC Laboratory for Molecular Cell Biology, UCL, London, UK; c Molecular Cell Biology of Autophagy, The Francis Crick Institute, London, UK

**Keywords:** AP4B1, AP4E1, AP4M1, AP4S1, ER-phagy, mAtg9, reticulophagy, SPG47, SPG51, swelling, varicosities

## Abstract

Adaptor protein (AP) complexes mediate key sorting decisions in the cell through selective incorporation of transmembrane proteins into vesicles. Little is known of the roles of AP-4, despite its loss of function leading to a severe early onset neurological disorder, AP-4 deficiency syndrome. Here we demonstrate an AP-4 epsilon subunit knockout mouse model that recapitulates characteristic neuroanatomical phenotypes of AP-4 deficiency patients. We show that ATG9A, critical for autophagosome biogenesis, is an AP-4 cargo, which is retained within the *trans*-Golgi network (TGN) *in vivo* and in culture when AP-4 function is lost. TGN retention results in depletion of axonal ATG9A, leading to defective autophagosome generation and aberrant expansions of the distal axon. The reduction in the capacity to generate axonal autophagosomes leads to defective axonal extension and *de novo* generation of distal axonal swellings containing accumulated ER, underlying the impaired axonal integrity in AP-4 deficiency syndrome.

**Abbreviations**: AP: adaptor protein; AP4B1: adaptor-related protein complex AP-4, beta 1; AP4E1: adaptor-related protein complex AP-4, epsilon 1; ATG: autophagy-related; EBSS: Earle’s balanced salt solution; ER: endoplasmic reticulum; GFAP: glial fibrillary acidic protein; GOLGA1/Golgin-97/GOLG97: golgi autoantigen, golgin subfamily a, 1; GOLGA2/GM130: golgi autoantigen, golgin subfamily a, 2; HSP: hereditary spastic paraplegia; LC3/MAP1LC3B: microtubule-associated protein 1 light chain 3 beta; MAP2: microtubule-associated protein 2; MAPK8IP1/JIP1: mitogen-acitvated protein kinase 8 interacting protein 1; NEFH/NF200: neurofilament, heavy polypeptide; RBFOX3/NeuN (RNA binding protein, fox-1 homolog [C. elegans] 3); SQSTM1/p62: sequestosome 1; TGN: trans-Golgi network; WIPI2: WD repeat domain, phosphoinositide interacting protein 2

## Introduction

Adaptor protein (AP) complexes have roles in the selection of transmembrane proteins (cargo) for inclusion into vesicles. AP complexes interact with sorting motifs within the cytoplasmic facing tails of cargoes, leading to their specific enrichment at sites on donor membranes. Upon motif recognition and binding to cargoes, AP complexes recruit coat proteins which assemble to generate free vesicles [1]. Of the 5 members of the AP complex family, AP-1 and AP-2 are the best understood thus far, functioning in clathrin-dependent sorting from the *trans*-Golgi network (TGN) and endocytosis from the plasma membrane, respectively. Assembling as hetero-tetramers, AP complexes require the presence of all subunits to maintain functionality [2–4]. Indeed, mutations in genes encoding all subunits of AP-4 (ϵ1; *AP4E1*, β1; *AP4B1*, μ1; *AP4M1* and σ1; *AP4S1*) have been identified as leading to a complex form of hereditary spastic paraplegia (HSP) termed AP-4 deficiency syndrome (henceforth AP-4 deficiency) [5,6]. AP-4 deficiency patients present with early-onset severe intellectual disability, absence of speech and progressive spasticity leading to para- or tetraplegia [7]. Thinning of the corpus callosum axonal tracts and ventriculomegaly are characteristic neuroanatomical features arising from white matter loss in AP-4 deficiency patients [5,7–10]. Despite this severe pathology little is known of AP-4 other than its localization to the TGN in cell lines [11,12], and mislocalization of AMPA receptors to autophagosomes in the axons of a mouse model lacking *ap4b1* [13]. The entire repertoire of cargoes sorted by AP-4 in neurons and the functional consequence of their altered handling and subsequent trafficking as a result of disruption of the AP-4 complex remain poorly understood.

Macroautophagy (henceforth autophagy) is the process by which organelles and macromolecules are recycled for the maintenance of cellular homeostasis. Autophagy can be simplified into 3 fundamental steps; induction, autophagosome biogenesis and lysosomal degradation. Progression through the pathway is in part mediated by the concerted recruitment of autophagy-related (ATG) proteins [14], the sequence and necessity of which are understood to be conserved in the neuron [15]. After the induction of autophagy, membrane elongation from sites on the endoplasmic reticulum (ER) forms a cup shaped phagophore which incorporates cytosolic components [16]. Enclosure of the expanding edges of the phagophore produces a double-membraned autophagosome, which can then fuse with late endosomes and lysosomes to form degradative autolysosomes [17].

Intact and efficient autophagy is of critical importance to post-mitotic cells, which cannot overcome proteotoxic burden through cellular division [18]. Neurons may be exceptionally susceptible to defects in autophagy due to their extreme architecture, particularly the axon which can extend for distances up to a meter from the soma [19,20]. Autophagosomes are constitutively generated in the axon, predominantly at its most distal extremities [21,22]. In order to maintain this localized biogenesis, machineries necessary for autophagosome generation must be delivered to the distal axon; and once completed, *de novo* generated autophagosomes retrogradely trafficked toward the soma for their clearance by resident lysosomes. The sole mammalian transmembrane ATG protein, ATG9A is thus of particular interest, as it relies upon vesicular sorting and trafficking mechanisms for its distribution. With its roles in phagophore extension and autophagosome maturation [23,24], effective sorting and delivery of ATG9A may be critical for the maintenance of constitutive generation of autophagosomes in the distal axon. Indeed, dysgenesis of the corpus callosum has recently been identified in a CNS-specific *atg9a* knockout-mouse model [25]. This defect in commissural axon crossing arising from loss of ATG9A is highly reminiscent of the thinning of the corpus callosum in AP-4 deficiency, raising the question of whether the recent identification of altered ATG9A sorting in cell lines lacking AP-4 function [26] is of relevance to the pathological features of AP-4 deficiency.

Here we identify neuroanatomical defects in an *ap4e1* knockout-mouse model mirroring those of AP-4 deficiency patients. We show that ATG9A is a neuronal AP-4 cargo, which is retained within the TGN *in vivo* and in culture as a consequence of the disruption of AP-4 assembly. TGN retention results in depletion of axonal ATG9A, reduction in the capacity of axonal autophagosome generation and aberrant accumulation of ER within swellings in the distal axon. This alteration in axonal autophagosome biogenesis likely underlies defective axonal extension and integrity when AP-4 function is lost, leading to axonal loss and the characteristic phenotypes of AP-4 deficiency syndrome.

## Results

### *Ap4e1* null mice recapitulate neuroanatomical features of AP-4 deficiency

**Figure 1.**
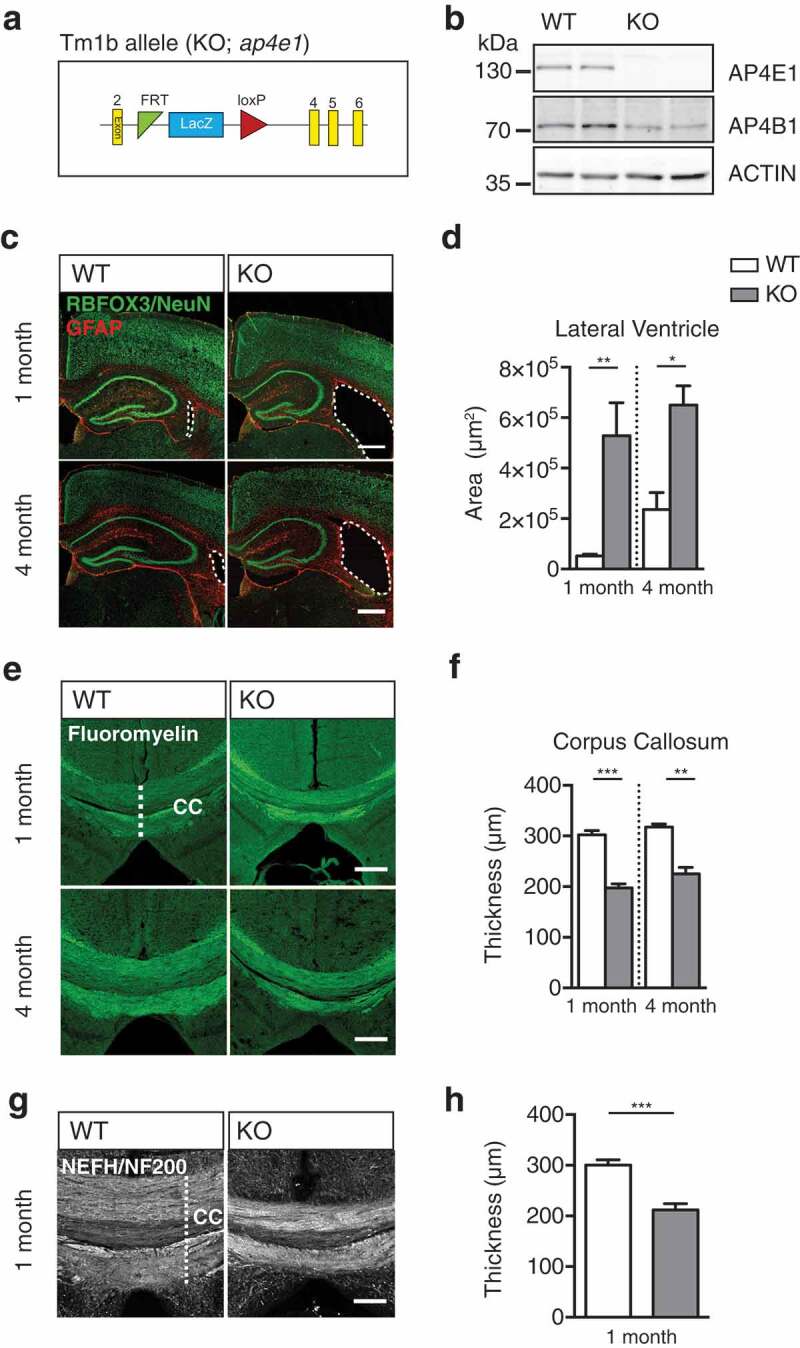
*ap4e1* null mice recapitulate neuroanatomical features of AP-4 deficiency. (**a**) Schematic of promoter driven *ap4e1* KO tm1b allele, showing removal of critical exon 3. (**b**) Western blot prepared from AP-4 line showing total loss of AP4E1 protein and concurrent loss of AP4B1 at protein level in *ap4e1* KO animals. (**c, d**) Sections prepared from animals at 1 and 4 month timepoints stained against RBFOX3/NeuN and GFAP showing lateral ventricular enlargement in KO. Scale bar: 200 μm. (D) Quantification of relative area of lateral ventricle (n = 3/5 animals 1 and 4 months). (**e, f**) Axons stained using FluoroMyelin at 1 month and 4 months showing thinning of corpus callosum tracts in KO. Scale bar: 200 μm. (**f**) Quantification of thickness of corpus callosum (n = 3 animals). (**g, h**) Commissural crossing axons at 1 month, stained against Neurofilament-200 (NEFH/NF200). Scale bar: 100 μm. (**h**) Quantification of thickness of corpus callosum (n = 5 animals). Quantified data expressed as mean ± SEM. Statistical analysis: Two-tailed unpaired Student’s t-test, *p < 0.05, **p < 0.01 and ***p < 0.001. CC – corpus callosum.

To elucidate the mechanisms underpinning the pathology in AP-4 deficiency, we first sought to characterize a mouse model developed by the International Mouse Phenotyping Consortium (IMPC) by targeting one of the 2 large subunits of the AP-4 complex [27]. Heterozygous mice carrying 1 copy of the targeting cassette (Figure 1(a)) were crossed, generating litter matched *Ap4e1*^+/+^ (WT) and *ap4e1*^−/-^ (KO) animals (Figure S1(a)). The AP4E1 subunit was confirmed to be entirely absent at the protein level in KO animals (Figure 1(b)), and levels of the AP4B1 subunit were reduced in tandem, indicating destabilization of the entire AP-4 complex [2–4], and thus loss of AP-4 complex assembly. Using a beta-galactosidase assay we showed the AP4E1 subunit to be expressed throughout all tissues during embryonic development (Figure S1(b,c)), and across regions in the adult brain (Figure S1(d)).

We next examined whether mice with loss of AP-4 function phenocopied the characteristic neuroanatomical features of AP-4 deficiency patients. To this end, we prepared sections from WT and KO mice and examined their brain morphology through staining of RBFOX3/NeuN and GFAP (Figure 1(c)). We found dramatic and specific enlargement of the lateral ventricles in KO animals by 1 month, and that this enlargement did not appear to progress in severity by 4 months (Figure 1(d), S1E; lateral ventricular area; 1 month: WT 52,216 ± 7,092 μm^2^, KO 528,509 ± 130,084 μm^2^, p = 0.0064; 4 month: WT 235,874 ± 67,610 μm^2^, KO 650,456 ± 76,361 μm^2^, p = 0.015; Figure S1(e); area of third ventricle: WT 297,649 ± 51,048 μm^2^, KO 278,361 ± 34,709 μm^2^, p = 0.78; area of fourth ventricle: WT 9,966 ± 2,333 μm^2^, KO 9,793 ± 1815 μm^2^, p = 0.96; t-test). Given this similarity to patients’ ventriculomegaly, we next examined whether KO mice additionally exhibited the characteristic thinning of the corpus callosum by staining brain sections using FluoroMyelin to reveal axon tracts (Figure 1(e)). Robust thinning of the corpus callosum axonal tracts was evident at 1 month in KO animals, which did not progress in severity by 4 months (Figure 1(f); corpus callosum thickness FluoroMyelin; 1 month: WT 302.5 ± 8.26 μm, KO 197.4 ± 8.25, p = 0.0008; 4 month: WT 317.7 ± 5.74 μm, KO 225.4 ± 12.52, p = 0.0026; t-test). We confirmed that this was as a result of the loss of axons by staining sections against the axon-resident neurofilament, NEFH/NF200 (Figure 1(g,h); corpus callosum thickness NEFH/NF200: WT 300 ± 10.7 μm, KO 212 ± 12.7 μm, p = 0.0007; t-test). In addition to the corpus callosum, thinning of the apical anterior commissure and external capsule axonal tracts was evident at 1 month in KO animals (Figure S1(f, g); anterior commissure area: WT 80,200 ± 12,050 μm^2^, KO 45,900 ± 4,950 μm^2^, p = 0.0272; external capsule length: WT 142.3 ± 6.2 μm^2^, KO 97.22 ± 4.29 μm^2^, p < 0.0001; t-test), whereas no alteration was found in the lateral optic tract (Figure S1(h); lateral optic tract: WT 38,500 ± 2716 μm, KO 37,140 ± 3,890 μm; p = 0.78).

The thinning of commissural axon tracts and enlargement of lateral ventricles led us to question whether these defects arise due to defective axon development, or through extensive neuronal cell loss. To address this, we quantified neuronal cell numbers within regions of KO brain at 1 and 4 months. We found that cortical neuronal numbers in KO were indistinguishable from WT animals both at 1 and 4 months (Figure S1(i); relative cortical neuronal cell number; 1 month: WT 1 ± 0.053, KO 1.053 ± 0.033, p = 0.44; 4 month: WT 1 ± 0.09, KO 0.94 ± 0.057, p = 0.63; t-test). Neither was there a detectable reduction in neuronal cell number in major regions of the hippocampus at 1 month (Figure S1(j, k); relative neuronal cell number; CA1: WT 1 ± 0.11, KO 1.05 ± 0.031, p = 0.72; CA3: WT 1 ± 0.077, KO 0.99 ± 0.057, p = 0.92; DG: WT 1 ± 0.059, KO 0.97 ± 0.093, p = 0.78; t-test). We did note however a small decrease in cell number in the CA1 region of the hippocampus at 4 months, whereas CA3 and DG regions were unaffected (Figure S1(j, l); relative neuronal cell number; CA1: WT 1 ± 0.037, KO 0.85 ± 0.031, p = 0.036; CA3: WT 1 ± 0.076, KO 0.79 ± 0.045, p = 0.082; DG: WT 1 ± 0.073, KO 0.91 ± 0.14, p = 0.59; t-test). To further examine whether there was evidence of neuronal cell death in KO brains, we investigated astrocytic proliferation in regions of the hippocampus. No increase in astrocyte resident GFAP immunoreactivity was evident at 1 month nor 4 months (Figure S1(j,m,n); relative GFAP immunoreactivity; 1 month CA1: WT 1 ± 0.11, KO 0.93 ± 0.098, p = 0.66; 1 month CA3: WT 1 ± 0.086, KO 1.02 ± 0.15, p = 0.91; 1 month DG: WT 1 ± 0.18, KO 0.94 ± 0.068, p = 0.77; 4 month CA1: WT 1 ± 0.055, KO 0.82 ± 0.086, p = 0.15; 4 month CA3: WT 1 ± 0.15, KO 0.97 ± 0.11, p = 0.9; 4 month DG: WT 1 ± 0.083, KO 0.82 ± 0.095, p = 0.22; t-test), indicating that there was no astrogliosis and thus no evidence of neurodegenerative cell death in KO animals. The identification of enlarged lateral ventricles and concurrent thinning of the corpus callosum are highly reminiscent of the characteristic features of AP-4 deficiency patients [7], supporting *ap4e1* KO mice as a model of AP-4 deficiency. We show that these anatomical defects are evident at 1 month and have no progressive element in our model by 4 months of age.

### ATG9A trafficking is affected in vivo in *ap4e1* KO mice

We next questioned what essential functions of AP-4 or its cargoes are lost that leads to the specific and severe axonal pathology evident in AP-4 deficiency. Intriguingly, the transmembrane protein ATG9A has been identified as a putative AP-4 interactor by mass spectroscopy [28] and recent work has highlighted that ATG9A has altered distribution in cell lines lacking AP-4 [26,29]. Given the localization of AP-4 to the TGN, and the necessity of sorting transmembrane ATG9A from the TGN to the distal axonal for local autophagosome generation; we sought to elucidate whether this alteration of ATG9A handling was evident *in vivo*, and thus potentially the mechanism underpinning the pathology in AP-4 deficiency.

**Figure 2.**
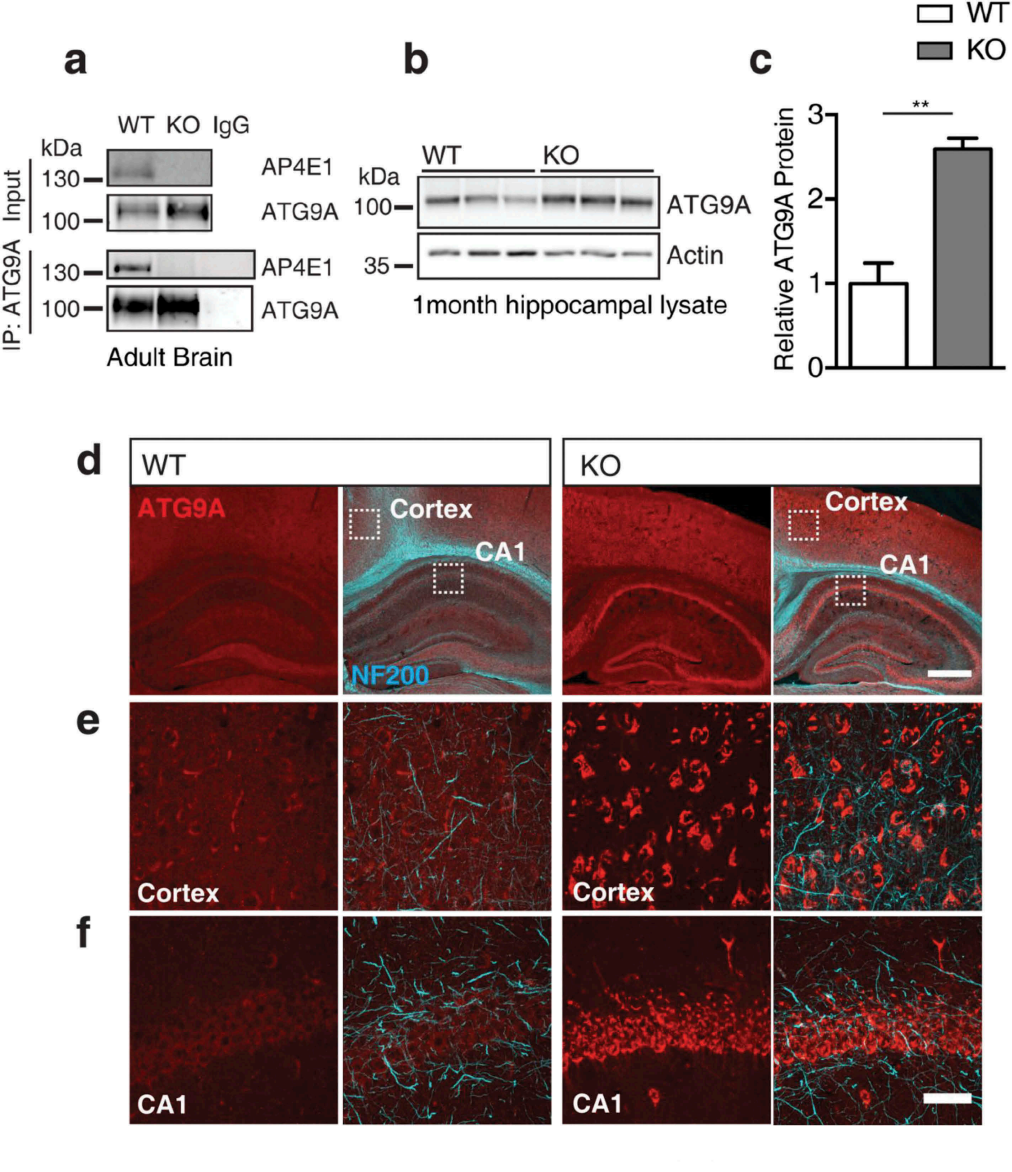
ATG9A handling is affected *in vivo* in *ap4e1* KO mice. (**a**) Endogenous co-immunoprecipitation of AP4E1 with ATG9A from mouse brain, showing interaction between AP-4 and ATG9A (n = 3 independent experiments). (**b**) Western blot of ATG9A in hippocampus at 1 month showing increase in KO animals. (**c**) Quantification of relative ATG9A protein (n = 3 animals). (**d**) Sections prepared from AP-4 line at 1 month stained against ATG9A and NEFH/NF200, showing increased immunoreactivity and accumulation of ATG9A within neuronal cell layers. Scale bars: 200 and 50 μm (**e**) High magnification showing accumulation of ATG9A within cortex and (**f**) CA1 of hippocampus. Scale bar: 20 μm (n = 5 animals). Quantified data is expressed as mean ± SEM. Statistical analysis: Two-tailed unpaired Student’s t-test, **p < 0.01.

Confirming the interaction between ATG9A and the AP-4 complex by Co-IP of ATG9A and AP4E1 from adult mouse brain (Figure 2(a)), we showed that ATG9A is an AP-4 cargo in the brain. Interestingly, in these immunoprecipitation experiments we also noticed that the protein level of ATG9A was robustly increased in our input samples. Preparing lysates from KO hippocampus at 1 month we confirmed the increase in ATG9A protein in KO brain (Figure 2(b,c); relative ATG9A protein: WT 1 ± 0.23, KO 2.33 ± 0.05, p = 0.0046; t-test), indicating that not only did ATG9A interact with AP-4, but its protein levels were affected by loss of AP4E1. Indeed, ATG9A was increased at protein level in all regions of the brain investigated (Figure S2(a)), and throughout other tissues including the heart, liver and lung in KO animals (Figure S2(b)). Investigating brain sections from mice at 1 month, we found not only a marked increase in the immunoreactivity of ATG9A in KO brain (Figure 2(d)), but notably that ATG9A appeared to accumulate within distinct structures in neuronal cell layers of the cortex and CA1 regions of KO brain (Figure 2(e,f)). Thus, it is evident that loss of AP-4 function dramatically alters the amount and localization of ATG9A *in vivo* in *ap4e1* KO animals, suggesting that ATG9A function may be affected in AP-4 deficiency.

### ATG9A accumulates within the TGN in *ap4e1* KO neurons

**Figure 3.**
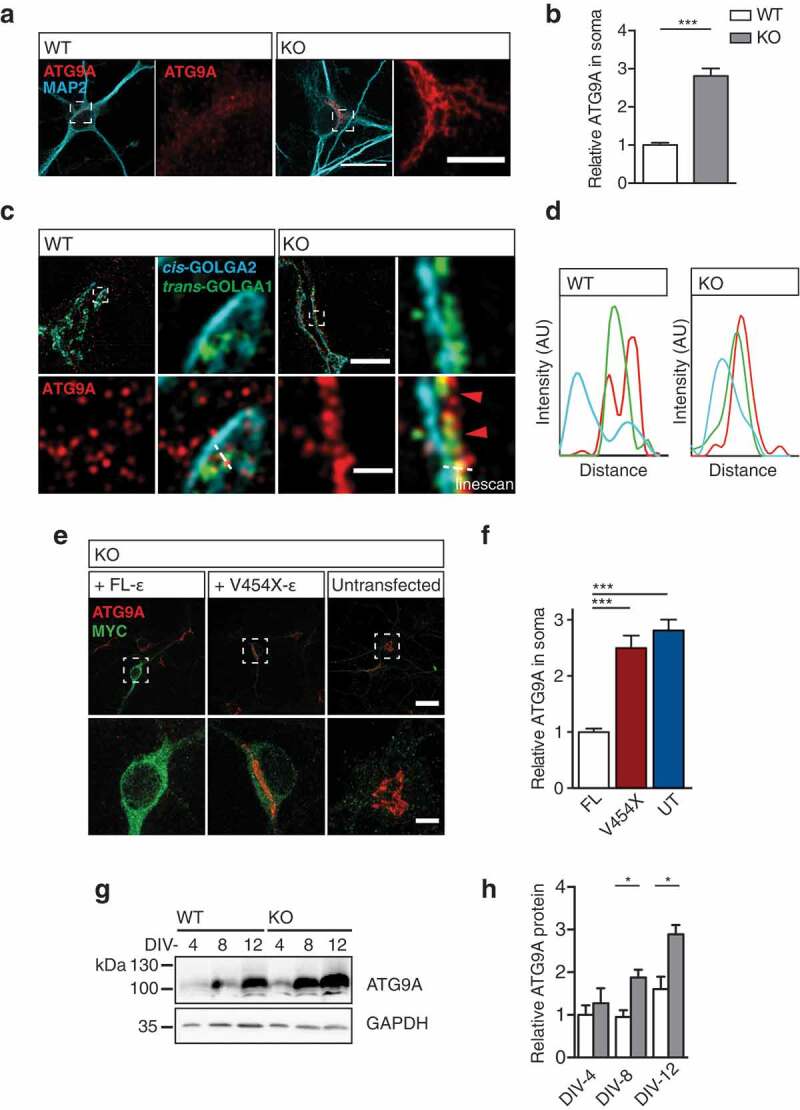
ATG9A accumulates within the TGN in *ap4e1* KO neurons. (**a**) DIV-8 cultured hippocampal neurons stained against ATG9A and MAP2. Inset panels show zoomed regions indicated by dashed boxes of cell body and accumulation of ATG9A. Scale bars: 20 μm; crop: 5 μm. (**b**) Quantification of relative ATG9A in neuronal soma (n = 40/20 neurons WT/KO). (**c**) SIM of DIV-8 cultured hippocampal neurons stained against ATG9A, *cis*-Golgi marker GOLGA2/GM130 and *trans*-Golgi marker GOLGA1/GOLG97. Dashed boxes indicate region in magnified panels, showing ATG9A overlapping GOLGA1/GOLG97 in KO. (**d**) Intensity linescans demonstrate ATG9A retention within the TGN in KO neurons. Scale bars: 5 μm; crop: 0.5 μm (n = 3 experimental repeats). (**e**) Reconstitution of AP-4 complex with expression of MYC-tagged FL AP4E1 and AP-4 deficiency associated AP4E1^V454X^ *Ap4e1* constructs (refer to Fig S3B). Dashed boxes indicate region in magnified panels. Scale bars: 5 μm; crop: 2 μm. (**f**) Quantification of relative ATG9A in soma of KO neurons in rescue conditions (n = 26/26/23 neurons FL AP4E1/AP4E1^V454X^/UT). (**g**) Western blot of lysates prepared from cortical neurons after 4, 8 and 12 days in culture probed against ATG9A. (**h**) Quantification of relative levels of ATG9A (n = 3/4 embryos WT/KO). Quantified data is expressed as mean ± SEM. Statistical analysis: (b, h) Two-tailed unpaired Student’s t-test, (f) Kruskall-Wallis test, *p < 0.05 and ***p < 0.001.

Given the accumulation of ATG9A in KO neurons *in vivo*, we sought to identify the compartment within which ATG9A was retained. To address this, we examined cultured hippocampal neurons, staining against ATG9A and the neuron specific dendritic marker, MAP2 (Figure 3(a)). Similar to our *in vivo* findings, endogenous ATG9A was increased in KO neurons (Figure 3(a,b); relative neuronal ATG9A signal: WT 1 ± 0.06, KO 2.8 ± 0.2, p < 0.0001; t-test), and other cell types including astrocytes (Figure S3(a); relative astrocytic ATG9A signal: WT 1 ± 0.11 KO 2.42 ± 0.27, p < 0.0001; t-test).

Given the known localization of AP-4 to the TGN in cell lines, we hypothesized that the sorting of ATG9A may be dependent on AP-4, and thus ATG9A may be retained within the TGN in neurons [26,29,30]. We firstly confirmed neuronal AP-4 localization at the TGN, through staining of endogenous AP4E1 and the TGN resident marker, GOLGA1/Golgin-97/GOLG97 (Figure S3(b)). Following this, we used a super-resolution approach (structured illumination microscopy, SIM) to clarify the ATG9A compartment in KO neurons. Staining the *cis* and *trans* faces of the Golgi network (GOLGA2, and GOLGA1 respectively), we found that ATG9A was specifically retained in the TGN of KO neurons (Figure 3(c,d)). In contrast, ATG9A exhibited a vesicular localization in WT neurons, highlighting the dramatic alteration in ATG9A sorting and localization when AP-4 function is lost.

To confirm that TGN retention of ATG9A arose as a result of failure of AP-4 dependent sorting, we opted to reconstitute the AP-4 complex through exogenous expression of the AP4E1 in KO neurons. We cloned N-terminally tagged AP4E1 (Figure S3(b)), and through transient transfection examined the effect upon ATG9A retention (Figure 3(e)). Reconstitution of the AP-4 complex with a full-length (FL) AP4E1 was able to entirely rescue ATG9A retention, and its levels in KO neurons. However, an AP4E1^V454X^ construct carrying a premature termination mutation identified in an AP-4 deficiency cohort [10], failed to restore ATG9A retention and levels in KO neurons (Figure 3(e,f); relative ATG9A in soma: FL 1 ± 0.06, AP4E1^V454X^ 2.48 ± 0.22, untransfected [UT] 2.81 ± 0.19, p < 0.0001 AP4E1^V454X^/UT to FL, NS between AP4E1^V454X^ and UT; Kruskall-Wallis). Thus, functional AP-4 assembly is necessary for the TGN exit of ATG9A both *in vivo* and in cultured neurons. Does this retention lead to the increased levels of ATG9A evident in KO animals? Preparing lysates from neurons at DIV-4, DIV-8 and DIV-12 we identify that the level of ATG9A increased progressively over time (Figure 3(g,h); relative ATG9A protein; DIV-4: WT 1 ± 0.22, KO 1.27 ± 0.35, p = 0.55; DIV-8: WT 0.95 ± 0.16, KO 1.88 ± 0.18, p = 0.014; DIV-12: WT 1.6 ± 0.29, KO 2.89 ± 0.22, p = 0.012; t-test), confirming that retention within the TGN results in the accumulation of ATG9A protein.

### Defective autophagosome maturation in *ap4e1* KO neurons

**Figure 4.**
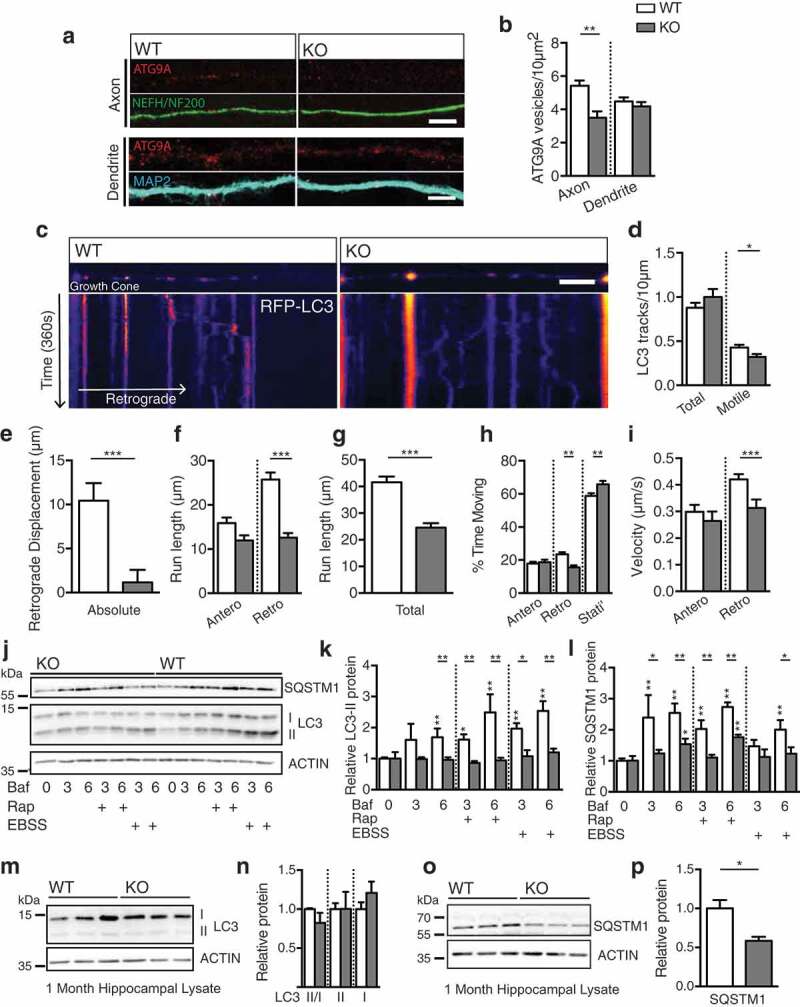
Defective autophagosome maturation in *ap4e1* KO neurons. (**a**) Endogenous ATG9A vesicles in axons and dendrites, stained using NEFH/NF200 and MAP2 markers respectively. (**b**) Quantification of ATG9A vesicles per 10 μm^2^ in axons and dendrites. Scale bar: 5 μm. (Axon; n = 19/12 WT/KO, Dendrite; 28/24 WT/KO). (**c**) Live imaging of nascent autophagosome biogenesis in distal most 75 μm of axon, showing aberrant autophagosome maturation in KO axons. Movies generated over 6 min from cultured hippocampal neurons at DIV-6/7 transfected with RFP-LC3. First frames and kymographs shown with pseudo-coloring of RFP-LC3 signal. X-axis scale bar: 10 μm, Y-axis represents time (1 px/1.5 s). Quantification of; (**d**) Total and motile RFP-LC3 tracks (**e**) absolute retrograde displacement, (**f**) anterograde and retrograde run length per motile autophagosome, (**g**) total distance travelled per motile autophagosome, (**h**) proportion of time spent stationary, moving anterogradely or retrogradely per motile autophagosome and (**i**) velocity of motile autophagosomes. (n = 227/117 motile autophagosomes from 46/36 neurons WT/KO). (Refer to Movies S1 and S2). (**j**) Autophagic flux assay in DIV-8 cultured cortical cultures. Neurons were treated in the presence of 100 nM bafilomycin for durations (in hours) indicated with either EBSS or 250 nM rapamycin to induce autophagy (n = 6 embryos). (**k**) Quantification of relative LC3-II and (**l**) SQSTM1/p62 levels relative to own 0 h control. (**m**) Western blot of lysates prepared from KO hippocampus at 1 month showing no alteration in endogenous LC3 levels nor processing. (**n**) Quantification of LC3-II:I ratio, LC3-II and LC3-I (n = 3 animals). (**o**) Blot of endogenous SQSTM1/p62 in KO hippocampus at 1 month showing reduction in AP-4 KO. (**p**) Quantification of relative protein level of SQSTM1/p62 (n = 3 animals). Quantified data is expressed as mean ± SEM. Statistical analysis: (**b, n and p**) Two-tailed unpaired Student’s t-test, (**d – i, k and l**) Mann-Whitney test, *p < 0.05, **p < 0.01 and ***p < 0.001.

Having elucidated the necessity of AP-4 for the sorting of ATG9A from the TGN, we next sought to understand what effect this retention had on the distribution and function of ATG9A in AP-4-deficient neurons. Staining dendritic and axonal compartments using MAP2 and NEFH/NF200 respectively, we found a specific reduction in the axonal pool of ATG9A (Figure 4(a,b); ATG9A vesicles per 10 μm^2^; axonal: WT 5.42 ± 0.32, KO 3.49 ± 0.39, p = 0.0009; dendritic: WT 4.47 ± 0.25, KO 4.18 ± 0.26, p = 0.43; t-test). Notably, dendritic ATG9A in KO was at parity with WT levels, whereas axonal ATG9A was reduced despite the near 3-fold increase in ATG9A within neuronal somas (Figure 3(b)). Together these results identify a clear reduction in the axonal pool of ATG9A, whereas dendritic ATG9A appears unaffected.

Given the known functions of ATG9A, what does this reduction in its axonal distribution mean for the capacity of autophagosome biogenesis within this compartment? To address this we examined nascent autophagosome maturation within the distal-most portions of the axon. Transient transfection of RFP-LC3 (MAP1LC3B), which associates with autophagosomes from early through to late maturation states, provided a robust marker for tracking autophagosomes throughout their lifespan [15]. Maturing autophagosomes initially exhibit bidirectional movement [21] prior to switching to robust dynein driven retrograde movement mediated by MAPK8IP1/JIP1 [31,32]. We found nascent autophagosomes in the distal WT axon to exhibit classical bi-directional motility with net retrograde displacement towards the neuronal soma (Figure 4(c), movies S1 and S2) [21]. In KO axons however, we found a reduction in the number of motile LC3-positive structures (Figure 4(c,d); RFP-LC3 structures; Total: WT 0.88 ± 0.35, KO 1 ± 0.47, p = 0.51; motile: WT 0.43 ± 0.2, KO 0.32 ± 0.16, p = 0.044; Mann-Whitney U), and that these nascent autophagosomes exhibited a reduced propensity to move retrogradely toward the soma, both in absolute retrograde displacement (Figure 4(c,e); absolute retrograde displacement: WT 10.43 ± 1.99 μm, KO 1.139 ± 1.42 μm, p < 0.0001; Mann-Whitney U test) and in individual retrograde runs, whereas anterograde movements were unaltered (Figure 4(f); anterograde run length: WT 15.89 ± 1.28 μm, KO 11.96 ± 1.16 μm, p = 0.686; retrograde run length: WT 25.75 ± 1.59 μm, KO 12.58 ± 1.055 μm, p < 0.0001; Mann-Whitney U). Autophagosomes in KO axons were less motile, with reduced total run length (Figure 4(g); total run length per autophagosome: WT 41.64 ± 2.11 μm, KO 24.54 ± 1.72 μm, p < 0.0001; Mann-Whitney U test), spent more time stationary (Figure 4(h): proportion time stationary: WT 58.75 ± 1.6%, KO 65.82 ± 2.077%, p = 0.0055; Mann-Whitney U) and less time moving retrogradely than in the WT axon (Figure 4(h); time moving anterogradely: WT 17.80 ± 1.17%, KO 18.66 ± 1.55%, p = 0.45; time moving retrogradely: WT 23.45 ± 1.29%, KO 15.52 ± 1.13%, p = 0.0003; Mann-Whitney U test). Importantly, anterograde motility and trafficking of RFP-LC3 structures in KO axons was entirely indistinguishable from that of WT in run lengths, in their proportion of time moving and velocity of individual runs (Figure 4(h); anterograde velocity; WT 0.29 ± 0.026 μm/s, KO 0.27 ± 0.035 μm/s, p = 0.38; mean retrograde velocity; WT 0.42 ± 0.02 μm/s, KO 0.31 ± 0.032 μm/s, p < 0.0001; Mann-Whitney U test). Given that anterograde movements were normal, we conclude that trafficking is not affected *per se*, but that there is a specific defect in retrograde movements of nascent autophagosomes that depends on LC3-lipidation state. Collectively these parameters identify a critical alteration in the maturation state of nascent autophagosomes in KO axons, owing to the reduced provision of axonal ATG9A as a result of the loss of AP-4 function.

We next examined whether alteration to autophagosome maturation impacted upon the autophagic capacity of KO neurons directly through investigation of autophagic flux in cultured neurons. DIV-8 neurons were treated in the presence of 100 nM bafilomycin A_1_ (BafA_1_) with 250 nM rapamycin (Rap) or starved in EBSS to induce autophagy (Figure 4(j)), and lipidated LC3 and SQSTM1/p62 levels examined by western blotting. A significantly reduced rate of LC3 lipidation was revealed in KO neurons, both basally, and with the induction of autophagy with rapamycin or EBSS (Figure 4(j,k); Table S1). Further, we found slowed SQSTM1/p62 accumulation in KO neurons during these treatments (Figure 4(j,l); Table S1). Interestingly, steady state levels of LC3 were unaffected in cultured KO neurons (Figure 4(j,k)), but also in hippocampal lysates prepared from animals at 1 month (Figure 4(m,n); relative LC3-II:I ratio: WT 1 ± 0.012, KO 0.82 ± 0.13, p = 0.24; relative LC3-II: WT 1 ± 0.077, KO 1 ± 0.22, p = 0.99; relative LC3-I: WT 1 ± 0.089, KO 1.21 ± 0.14, p = 0.29; t-test), in agreement with data recently reported [30]. Further, we found a robust decrease in SQSTM1/p62 in KO animals at 1 month (Figure 4(o,p); relative SQSTM1/p62 protein: WT 1 ± 0.11, KO 0.59 ± 0.05, p = 0.025; t-test), similar to that reported in *ap4b1* null mice [13]. The level of WIPI2 in the KO was at parity with WT animals (Figure S4(a,b); relative WIPI2 protein: WT 1 ± 0.066, KO 1.16 ± 0.12, p = 0.32; t-test) and we found no accumulation of ubiquitinated proteins in KO brain (Figure S4(c,d); relative ubiquitin protein: WT 1 ± 0.14 KO 1.11 ± 0.05, p = 0.49; t-test). Together these results indicate that AP-4 dependent ATG9A sorting is required for proper induction of autophagy in neurons, and to maintain autophagosome generation in the distal axon.

Using endogenous WIPI2 as a marker of early sites of autophagosome generation [33], we investigated autophagosome generation within neuronal somas. We identified a reduction of WIPI2 sites in KO somas (Figure S4(e,f); relative number of WIPI2 puncta: WT 1 ± 0.075, KO 0.74 ± 0.094, p = 0.031; Mann-Whitney U) and a reduction in the number of autophagosomes in KO neuronal somas, as evidenced by a reduction in the number of LC3 puncta (Figure S4(e,f); relative number LC3 puncta: WT 1 ± 0.097, KO 0.71 ± 0.12, p = 0.011; Mann-Whitney U). Indeed, despite this reduction in the numbers of puncta of both WIPI2 and LC3 in the soma, neither exhibited alteration in their size (Figure S4(e,g); size of LC3 puncta: WT 0.084 ± 0.0044 μm^2^, KO 0.078 ± 0.006 μm^2^, p = 0.17; size of WIPI2 puncta: WT 0.084 ± 0.0049 μm^2^, KO 0.094 ± 0.0085 μm^2^, p = 0.42; Mann-Whitney U), and LC3 associated with WIPI2 in KO somas indistinguishably from WT (Figure S4(e,h); proportion LC3 associated with WIPI2: WT 13.5 ± 2.3%, KO 15.3 ± 3.77, p = 0.96; Mann-Whitney U). Thus, sites of biogenesis in the soma as marked by WIPI2 appear functional but are reduced in number leading to a reduction in *de novo* autophagosome generation and thus flux. Together, these results indicate a slowing of autophagosome biogenesis through altered ATG9A sorting in *ap4e1* KO, resulting in fewer autophagosome being generated. However, once generated autophagic degradation in KO neurons appears to be normal.

### Axon specific defects in *ap4e1* KO neurons

**Figure 5.**
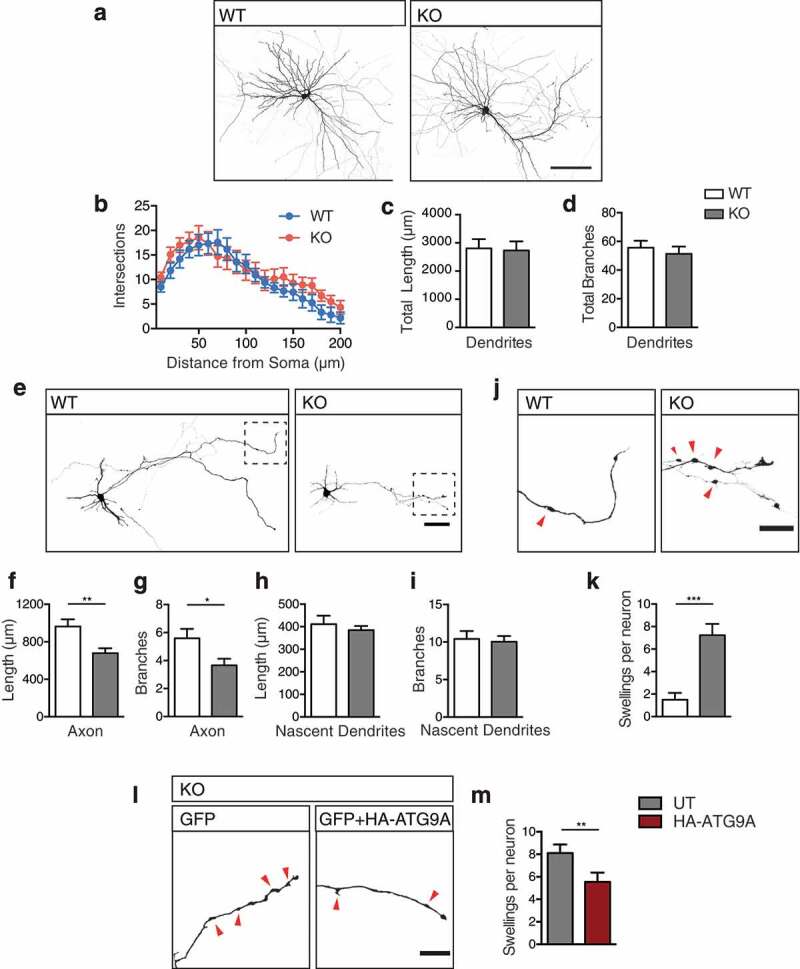
Axon specific defects in *ap4e1* KO neurons. (**a**) Cultured GFP-filled DIV-14 hippocampal neurons stained against GFP showing neuronal morphology. Scale bar: 100 μm. (**b**) Analysis of dendritic complexity using 10 μm concentric sholl intersections. Quantification of (**c**) total dendritic length and (**d**) total branches per neuron (n = 12/17 neurons WT/KO). (**e**) Cultured GFP-filled DIV-4 hippocampal neurons stained against GFP revealing neuronal morphology and entire axonal extension from individual neurons. Scale bar: 50 μm. (**f**) Quantification of total axonal length and (**g**) axonal branches. (n = 22/18 neurons WT/KO). (**h**) Quantification of nascent dendritic processes in total length and, (**i**) total branches (n = 22/27 neurons WT/KO). (**j**) Inset magnified panel from (**e**) of distal axonal regions, red arrows indicating axonal swellings. Scale bar: 20 μm. (**k**) Quantification of number of swellings per neuron (n = 20/17 neurons WT/KO). (**l**) Distal axonal regions of KO neurons transfected with GFP alone, or in combination with HA-ATG9A, showing a reduction in the number of axonal swellings upon expression of ATG9A, scale bar: 20 μm, (**m**) Quantification of number of swellings per neuron (n = 29/26 neurons WT/KO). Quantified data is expressed as mean ± SEM. Statistical analysis: (b) Two-way ANOVA with Bonferroni post-hoc test, (**c, d, f, h and i**) Two-tailed unpaired Student’s t-test, (**g, k and m**) Two-tailed Mann-Whitney U test, *p < 0.05, **p < 0.01 and ***p < 0.001.

Does the reduction in the capacity of autophagosome generation underlie the overt axonal pathology evident in AP-4 deficiency? To answer this, we investigated the integrity of hippocampal neurons in our AP-4 deficiency model. Through transient transfection of GFP we revealed the entire morphology of cultured hippocampal neurons (Figure 5(a)) and examined their integrity after 14 days in culture. We identifed no alteration in dendritic complexity (Figure 5(b); number of intersections per 10 μm sholl: NS; two-way ANOVA), total dendritic length (Figure 5(a,c); total dendritic length: WT 2807 ± 326.1 μm, KO 2730 ± 324.2 μm, p = 0.87; t-test) nor branch number (Figure 5(a,d); total dendritic branches: WT 55.6 ± 4.9, KO 51.3 ± 5.1, p = 0.56; t-test). Using Golgi silver staining of slices prepared from our model at 4 months, we reconstructed entire dendritic arbors of CA1 hippocampal neurons (Figure S5(a)). Similarly to our findings in culture, we found no significant alteration in the complexity of apical (Figure S5(b); apical dendritic length per 10 μm; NS; two way ANOVA) nor basal dendrites (Figure S5(c); basal dendritic length per 10 μm; NS; two way ANOVA), total lengths of both apical and basal dendrites (Figure S5(d); total dendritic length; apical: WT 1529 ± 206 μm, KO 1732 ± 312 μm, p = 0.6; basal: WT 1037 ± 125 μm, KO 1058 ± 133 μm, p = 0.91; t-test), nor numbers of dendritic branches in KO neurons (Figure S5(e); total dendritic branches; apical: WT 15.9 ± 2.1, KO 15.2 ± 2.7, p = 0.85; basal: WT 12.4 ± 1.4, KO 11.3 ± 1.5; p = 0.6; t-test). Together we show that dendritic morphology is unaffected by the loss of AP-4 function both *in vivo* and cultured neurons.

We next examined whether the reduced capacity of axonal autophagosome maturation gives rise to a specific alteration to axonal integrity and thus the axonal pathology of AP-4 deficiency. Investigating cultured neurons at DIV-4, after the specification of the axonal process, we were able to examine the entirety of the axonal extension of an individual neuron. We identified decreased axon length in KO neurons (Figure 5(e,f): total axonal length; WT 963.5 ± 76.2 μm, KO 679.2 ± 51.9 μm, p = 0.0055; t-test), and in axon branching (Figure 5(e,g): number of axonal branches: WT 5.6 ± 3.2, KO 3.7 ± 1.9, p = 0.023; Mann-Whitney U test). Despite this, at this age in culture nascent dendritic processes of KO neurons were unaltered in their length (Figure 5(e,h); nascent dendritic length: WT 412 ± 37.6 μm, KO 385 ± 19.1 μm, p = 0.5; t-test) and branching (Figure 5(i): nascent dendritic branches: WT 10.4 ± 1.1, KO 10.0 ± 0.77, p = 0.772; t-test), highlighting that axonal extension is specifically affected. This reduction in axonal extension and branching may underpin the ventriculomegaly and thinning of the corpus callosum characteristic of AP-4 deficiency.

#### Distal generation of axonal swelling, comprised of ER accumulations in AP-4 deficiency

**Figure 6.**
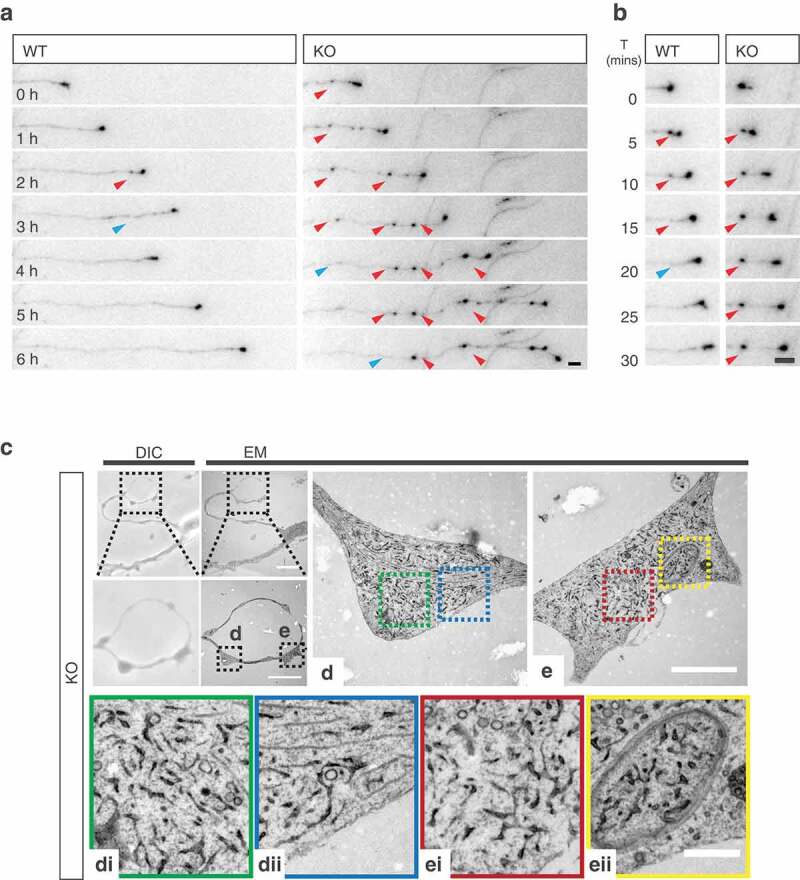
Distal generation of axonal swelling comprised of ER accumulations in AP-4 deficiency. (**a**) Long-term imaging of DIV-6 hippocampal neurons transfected with GFP enabling tracking of growing axons. Axonal swellings are *de novo* generated distally in close proximity to the growth cone in KO axons, indicated by red arrows. Swellings disassemble over time, as indicated by blue arrows (Refer to Movies S3 and S4). Scale bar: 5 μm. (**b**) Swellings formed in WT axons disassemble rapidly, whereas KO swellings persist at their site of deposition for dramatically longer durations (formation indicated by red arrow, disassembly by blue arrow) (Refer to movies S5 to S8). Scale bar: 5 μm. (**c**) Cultured DIV-4 hippocampal neurons were identified by DIC (b) for CLEM, showing correlation of axonal region with swellings, scale bars: 20 μm, 10 μm. Please refer to Fig S6B for WT example. Individual swellings (**d**) and (**e**) shown at higher magnification, scale bar: 2 μm. Cropped regions of (**d)** indicated by blue and green dashed boxes, (**di**) and (**dii**) respectively, showing accumulation of ER within axonal swelling. (**e**) Swelling showing incorporation of ER into double membraned autophagosome within axonal swelling, cropped regions indicated by red and yellow dashed boxes, (**ei**) and (**eii**). Scale bar: 0.5 μm. (n = 3 experimental repeats).

In examining axonal extension in culture, we noted that KO axons exhibited dramatic distal swellings (Figure 5(e,j,k); axonal swellings: WT 1.5 ± 0.61, KO 7.2 ± 1, p < 0.0001; Mann-Whitney U test), that were not seen in dendritic processes. These swellings were also evident in more mature cultures (Figure S5(f,g); axonal swellings per 100 μm: WT 0.23 ± 0.04, KO 1.1 ± 0.2, p = 0.005; t-test). Do these arise directly as a result of reduced axonal ATG9A (Figure 4(a)), or is there contribution from other AP-4 cargoes? To address this, we attempted to artificially drive increased axonal ATG9A through transient exogenous expression of HA-ATG9A (Figure S5(h)). We found that expression of HA-ATG9A for 2 days was sufficient to increase axonal delivery of ATG9A in KO axons, presumably through inclusion of exogenous ATG9A into other axonally-targeted vesicles (Figure S5(h,i); relative axonal ATG9A: untransfected (UT) 1 ± 0.19 HA-ATG9 4.8 ± 0.68, p < 0.0001; Mann-Whitney U test). As a result of this transient increase in axonal ATG9A, we found a significant reduction in the number of axonal swellings in KO axons (Figure 5(l,m); swellings per neuron: UT 8.1 ± 0.76, HA-ATG9 5.5 ± 0.83, p = 0.0075; Mann-Whitney U test), supporting ATG9A as the primary contributor to axonal swelling in KO neurons. Intriguingly, distal axonal swellings have previously been identified in mouse models ablated of ATG5 and ATG7 function [34,35]; ATG5, ATG7 and ATG9A have roles in autophagosome biogenesis post the initiation machinery [14,36], suggesting that axonal swellings may arise where autophagosome biogenesis has been initiated, but is stalled at this stage. To elucidate the nature of axonal swelling in KO neurons, we examined growing axons using long-term imaging of neurons transiently transfected with GFP for durations of 6 h (Figure 6(a), movies S3, S4). Strikingly, this revealed that swellings are generated *de novo* in *ap4e1* KO axons, emerging either directly from, or in close proximity to the advancing growth cone. These generated swellings were non-motile and persisted for durations in the range of hours (Figure 6(a), movies S3, S4). Interestingly, we found that growing WT axons also have the propensity to generate smaller, more transient swellings (Figure 6(b), S6A movies S5, S6, S7, S8), which disassembled rapidly when compared to those of KO axons.

We next addressed what these *de novo* generated distal swellings comprised of using a correlative light and electron microscopy (CLEM) approach. Identifying individual swellings in cultured neurons (Figure 6(c), S6(b)), we found that swellings in KO axons contained aberrantly accumulated ER (Figure 6(b-d)); whereas expanded portions of distal WT axons were largely devoid of ER (Figure S6(b)). Intriguingly, we also identified that these accumulations were incorporated into autophagosomes in KO axons (Figure 6(e), 6Eii, S6(e)). These findings, in combination with our data showing impaired autophagosome generation and autophagic flux, suggest that the accumulation of ER membranes in the axonal swellings of *ap4e1* KO axons is due to the reduced capacity of autophagosome generation where AP-4 dependent axonal ATG9A provision is reduced.

## Discussion

Despite mounting evidence implicating impaired autophagy in neurodevelopmental and neurodegenerative disease [37–42], autophagy remains poorly understood mechanistically in the neuron. The post-mitotic nature of the neuron, its extreme architecture, and limited capacity to upregulate autophagy [43] underlies its increased vulnerability to impaired autophagic flux. Here we show that failure of AP-4 mediated ATG9A sorting from the TGN depleted axonal ATG9A, impairing axonal autophagosome biogenesis. This resulted in aberrant accumulation of ER in *de novo* generated distal axonal swellings, likely underpinning the reduction in axonal extension, and neuroanatomical defects evident in our AP-4 deficiency mouse model. We provide evidence toward pathology in AP-4 deficiency arising through this specific reduction in the capacity of autophagic clearance in the axon.

In the present study, we identified neuroanatomical defects mirroring those of AP-4 deficiency patients. We show that these arose developmentally in our *ap4e1* KO mouse; thinning of the corpus callosum and lateral ventricular enlargement were evident by 1 month of age, and did not progress in severity by 4 months. Further, we show that these features did not result from neurodegenerative cell loss, as evidenced by the distinct lack of astrogliosis in KO animals at 4 months. Indeed, gross anatomy of KO mice was normal throughout development and into adulthood, despite their prominent neuroanatomical defects. We find that the thinning of axonal tracts *in vivo* arose as a result of the loss of axonal density; hippocampal neuron cultures revealed reduced axonal extension and branching at the level of the individual neuron. Strikingly however, the complexity and integrity of the dendritic arbors of hippocampal neurons were entirely indistinguishable from WT. The overt axonal pathology in AP-4 deficiency likely results from the specific depletion of axonal ATG9A in KO neurons; ATG9A being dramatically TGN retained where AP-4 is lost. We clarify that this is a feature of AP-4 deficiency by showing that functional AP-4 is required for the exit and sorting of ATG9A in neurons. Reconstitution of the AP-4 complex through exogenous expression of AP4E1 in KO neurons rescued ATG9A retention, whereas a pathological mutation carrying subunit was unable to do so [10]. Together, our findings reveal a critical role for AP-4 in ATG9A sorting in the neuron, highlighting the importance of axonal distribution of ATG9A for the maintenance of axonal integrity.

Maintenance of constitutive distal autophagosome biogenesis in the axon requires that the delivery of newly synthesized cargoes from the soma is tightly balanced with efficient clearance from this compartment. We show here that where the provision of ATG9A to the axon is reduced, the resulting slowing of autophagosome maturation lead to *de novo* generation of distal axonal swellings. These swellings comprised of expanded portions of ER are transient in their nature, being consumed through incorporation into autophagosomes. Swellings are immobile, persisting at their sites of formation for their duration. Thus, their collapse is not accounted for by re-distribution of constituent ER to more distally generated swellings, but likely through incorporation into autophagosomes. Together these results implicate a potential reduction in the capacity of axonal clearance of ER (reticulophagy), in the pathology of our model through the failure of AP-4 mediated ATG9A sorting. During the preparation of this manuscript we became aware of a study also identifying similar neuroanatomical defects in *ap4e1* KO mice [30]. Interestingly, the authors identify that over-expression of aggregate prone n-terminal fragments of huntingtin leads to its accumulation within axonal swellings. The identification herein of the dynamic nature of axonal swelling formation through slowing of autophagosome maturation as a result of reduced provision of axonal ATG9A provides the mechanism for this accumulation, and further adds to the evidence of a reduced capacity of autophagic clearance from the axon.

Distal axonal swellings are emerging as a hallmark in mouse models ablated of proteins critical for early stages of autophagosome biogenesis: *atg5, atg7, atg9a* [25,34,35], and are reported in multiple models of HSP [44–46]. Aberrant accumulation of ER is also found within the axonal swellings *of atg5* null mice [34], and ER expansion is evident where ATG5 or BECN1 are silenced [47]. Moreover, factors critical for ER shaping and remodeling including ATL1, REEP1, SPAST, RTN2 are implicated in over 60% of all HSP cases [48–51], and evidence is emerging that ATL1-dependent remodeling is necessary for selective autophagosomal incorporation of ER [52]. Given that autophagosomes form from ER sites [15,16], and ER resident proteins modulate autophagosome biogenesis [53] it remains to be ascertained in our model whether ER accumulated due to the reduction in the capacity to consume it [47,54,55]; or whether the reduced capacity of autophagosome biogenesis leads to reduced incorporation of ER into the autophagosomal membrane itself. Together, our model adds to the evidence implicating ER shaping, remodeling and additionally reticulophagy in the maintenance of axonal integrity in HSP [51,56–60].

70% of complex HSP patients presenting with progressive spasticity, intellectual impairment and thin corpus callosum are accounted for by mutations in SPG11/spatacsin and ZFYVE26/spastizin [6], both having roles in autophagosome maturation and endolysosomal function [57]. Thin corpus callosum has also been identified in autophagy-related CNS specific KO mouse models including *ulk1 ulk2* double knockout and *atg9a* [25,61], and axonal extension is concomitantly reduced in neurons cultured from these lines [25,56,62,63]. Given this striking similarity to *ap4e1* KO mice, we provide evidence towards thinning of the corpus callosum in AP-4 deficiency arising developmentally through defective axonal extension as a result of reduced axonal autophagosome biogenesis. Constitutive knockout of *atg5, atg7, atg9a* and *ulk1/2* double knockout leads to peri-natal lethality [64–68], whereas AP-4-deficient mice, and indeed patients, survive into adulthood. Importantly, in the present study we show that autophagosome biogenesis was slowed, but not entirely blocked. Sites of autophagosome maturation as marked by WIPI2 were reduced in the soma, but appear functional, giving rise to fewer autophagosomes. This remaining autophagic capacity was able to sustain dendritic integrity even by 4 months *in vivo*, whereas axonal defects manifested by 4 days in culture. Given the progressive nature of ATG9A accumulation in KO neurons, we speculate that increased ATG9A resident within the TGN membrane leads to its stochastic incorporation into vesicles mediated by other carriers. As a result, sufficient vesicular ATG9A is delivered somatodendritically in KO neurons to maintain effective autophagy within this compartment, whereas axonally destined vesicular delivery is reduced. Indeed, in *C. elegans* where AP-4 is not evolutionarily conserved [69], axonal delivery of ATG9A is also critical for autophagosome biogenesis and axon outgrowth [70].

Interestingly, we were able to partially rescue axonal swelling in KO neurons through overexpression of exogenous ATG9A, which increased axonal delivery presumably through inclusion of ATG9A into other axonally targeted vesicles at the TGN. Thus, promoting axonal ATG9A delivery from the TGN represents an intriguing therapeutic possibility either through increased ATG9A biosynthesis or manipulation of the recently identified AP-4 cargo adaptor RUSC2 [29]. Whilst we cannot entirely rule out whether other unidentified AP-4 cargoes contribute to the alterations in autophagosome generation evident in the axon, the limited AP-4 cargo repertoire emerging in recent studies [29] and the known roles of ATG9A make it the prime candidate in our AP-4-deficiency model.

In sum, we reveal a critical role of AP-4 in sorting ATG9A from the TGN in neurons. Impairment of this function as evident in the AP-4 deficiency model leads to accumulation of ATG9A within the TGN *in vivo* and in culture, leading to a specific reduction to the axonal delivery of ATG9A. As a result, axonal autophagosome biogenesis is defective, likely underlying the axonal defects evident in *ap4e1* KO mice. Together, our findings provide evidence towards a mechanism of pathology in AP-4 deficiency.

## Materials and methods

### Animals

*ap4e1* knockout (*ap4e1*^−/-^, C57BL/6J-*Ap4e1^[tm1b{KOMP}Wtsi^*^]^, KO) were generated using the knockout-first tm1b allele system [71] by the International Mouse Phenotyping Consortium (IMPC) MRC Harwell. Animals were maintained under controlled 12:12 h light-dark cycles at a temperature of 20 ± 2°C with food and water ad libitum. Genotyping was carried out using the following primers; AP4E1-5arm-WTF: GCCTCTGTTTAGTTTGCGATG, AP4E1-Crit-WTR: CGTGCACAGACAGGTTTGAT and 5mut-R1: GAACTTCGGAATAGGAACTTCG. Littermate matched controls were used for primary neuronal cultures and immunocytochemistry experiments. All experimental procedures were in accordance with UCL institutional animal welfare guidelines, and under the UK Home Office licence in accordance with the Animals (Scientific Procedures) Act 1986.

### Antibodies and DNA constructs

*Antibodies*: For immunocytochemistry (ICC), Immunohistochemistry (IHC) and western blotting (WB) antibodies were used with the following dilutions; ACTB/actin (Sigma, A2066; WB: 1:1,000), AP4B1 (Atlas Antibodies, HPA028652; WB: 1:200), AP4E1 (BD Biosciences, 612,018; WB: 1:300, ICC: 1:250), ATG9A (rabbit, STO-219; WB: 1:2000, IF: 1:2000 [72]) and rabbit ATG9A; (Abcam ab108338; WB: 1:1000, IF: 1:1000), ATG9A (Hamster 14F2 8B1; IF: 1:500 [72]), GAPDH (Abcam ab8245; WB: 1:10,000), GFAP (Dako, Z0334; ICC: 1:300), GFP (Nescalai Tesque 04404–84; ICC: 1:1000), GOLGA2/GM130 (BD Biosciences 610,822; ICC: 1:1000), GOLGA1/Golgin-97 (Cell Signaling Technology, 13,193; ICC: 1:250), HA (12CA5 hybridoma; WB: 1:200, ICC: 1:100), LC3B (Abgent AP1802a; WB: 1:250), LC3B (Cell Signaling Technology D-11 3868S; IF: 1:250), MAP2 (Synaptic Systems 188–004, ICC: 1:500), RBFOX3/NeuN (Chemicon International MAB377; IHC: 1:300), NEFH/NF200 (Abcam ab4680; IF: 1/500 IHC: 1/500), MYC (NeuroMab 9E10; WB: 1:100, ICC: 1:100), ubiquitin (Enzo Biosciences FK2; WB: 1/500) and WIPI2 (Ms Monoclonal; WB: 1:250, ICC: 1:250 [73]). HRP-conjugated anti-mouse/rabbit antibodies were used for western blotting at 1:10,000 (Jackson Laboratories, 1,706,516/1,706,515). Alexa Fluor-conjugated secondary antibodies for ICC, IHC and super-resolution imaging were used as follows; anti-chicken 405 and 647 (Abcam, ab175675 and ab150175) anti-guinea pig 405 and 647 (Abcam, ab175678 and ab150187 anti-mouse 488 and 647 (Jackson immunoresearch, 715–545-151-JIR and 715–605-151), anti-rabbit 555 and 647 (Abcam, ab1500074 and ab150063). Anti-Armenian hamster conjugated to Cy3 was used for super-resolution imaging (Jackson immunoresearch, 127–165-160).

*DNA Constructs*: CAG-GFP (Addgene, 16,664; deposited by F Gage), pmRFP-LC3 [74] (Addgene, 21,075; deposited by T Yoshimori). Full-length N-terminally MYC-tagged AP4E1 was generated by cloning the coding sequence of *Ap4e1* (Cusabio; CSB-CL890772HU, cDNA clone MGC: 163,338) into pRK5-Myc [75]. AP4E1^V454X^-ϵ was then generated by reverse mutagenesis methods replicating the reported 2 nucleotide insertion leading to frameshift induced premature stop at V454 [10]. HA-ATG9A was kindly gifted by the Tooze laboratory [72].

### Brain lysate preparation for co-immunoprecipitation

*Lysate Preparation*: Brains to be used for co-immunoprecipitation were removed from animals and homogenized in ice cold HEPES buffer (50 mM HEPES [GIBCO, 15,630–056], 0.5% Triton X-100 [Sigma Aldrich T-8787], 150 mM NaCl [Fisher Scientific, 11,904,061], 1 mM EDTA [Sigma Aldrich E5134], 1 mM PMSF [Panreac AppliChem, A0999,0025], 50 μl antipain, pepstatin and leupeptin [Peptide Institute, 4062, 4367, 4041] in ddH_2_O). Lysate was solubilized by rotation for 2 h at 4°C prior to ultracentrifugation at 80,000 g for 40 min. Protein content determined using a BCA assay kit (Promega, PI-23,225).

*Co-Immunoprecipitation*: Brain lysate (5 mg) was incubated with 1 μg of antibody in HEPES buffer for 12 h at 4°C with rotation, and 1 μg IgG control (rabbit; Thermo Fisher, 02–6102) was incubated with WT brain lysate in tandem. Input samples were incubated in the same manner at all steps as immunoprecipitation samples. Protein A agarose beads (Generon, PC-A25) were added for 4 h to IP samples, beads washed in HEPES buffer and suspended in protein sample buffer (150 mM Tris, pH 8 [Sigma Aldrich, 93,352], 6% SDS [Fisher Scientific, 10,090,490], 300 mM DTT [Melford, MB1015], 30% glycerol [Fisher scientific, 10,021,083], 0.3% bromophenol blue [Sigma Aldrich, B0126]) and heated to 95°C for 7 min prior to SDS-Page and western blotting.

### Brain and tissue lysate preparation; SDS-PAGE and western blotting

*Lysate Preparation*: Brains and tissues to be used for western blotting were removed from animals and snap frozen at −80°C. For preparation of lysates, brains and tissues were defrosted, relevant regions dissected and kept on ice throughout. Tissue was homogenized by sonication in lysis buffer (50 mM HEPES, pH 7.5, 0.5% Triton X-100, 150 mM NaCl, 1 mM EDTA, 1 mM PMSF, antipain/pepstatin/leupeptin as above), and debris pelleted at 38,000 x g for 10 min at 4°C. Lysate protein content was determined using a commercial BCA assay kit (Promega, PI-23,225) and samples denatured for 7 min at 95°C in protein sample buffer. Samples were stored at −80°C.

*SDS-PAGE and western blotting*: Protein lysate (20–40 μg) was separated by SDS-PAGE using Xcell Minicell II systems (Invitrogen, EI0002) and transferred onto nitrocellulose (GE healthcare, 10,600,003) or 0.45-μm pore PVDF (for LC3; GE Healthcare, 11,330,744). Membranes were blocked in milk (4% non-fat milk powder [Marvel, S0942], 0.05% Tween-20 [National Diagnostics, EC-607]) in PBS (137 mM NaCl [Fisher Scientific, 10,428,420], 2.7 mM KCl [Merck, 104,936], 10 mM Na_2_HPO_4_ [Merck, 106,586], 1.8 mM KH_2_PO_4_ [Merck, 529,568]), pH 7.4, 3% BSA [Sigma Aldrich, 05482-100G] for LC3 and AP4E1 for 1 h and incubated with primary antibodies at empirically determined dilutions as above overnight with agitation at 4°C. Membranes were then washed, secondary HRP-conjugated antibodies applied in milk at 1:10,000 and after a final washing steps bands visualized by application of ECL substrate (Millipore Luminata Crescendo, WBLUR0500) and imaging using a CCD based system (Quant LAS 4000, GE Healthcare). Densitometric analysis was performed using FIJI software (NIH).

### Beta-galactosidase staining of embryos, brains and sections

*Embryos and embryonic brains*: Embryos to be stained with X-gal were removed at developmental stages E12.5, E13.5 and E15.5. Due to impermeability of embryos at E15.5, brains were removed from animals and stained similarly to whole embryos. Embryos and brains were washed in PBS briefly, fixed in 4% PFA (Sigma-Aldrich, P6148-500G) for 90 min and stained at room temperature (RT) for 18 h (5 mM EGTA [Sigma-Aldrich, E4378], 2 mM MgCl_2_ [Melford, M0535], 0.2% gluteraldehyde [Sigma-Aldrich, G5882], 0.4% PFA, 0.01% DOC [Sigma-Aldrich, D6750], 0.02% NP40 [Millipore Calbiochem, 492,018] in PBS). Subsequently, embryos and brains were washed in PBS and serially dehydrated in increasing concentrations of EtOH (50–100%), and stored at 4°C in 100% EtOH.

*Adult brain X-Gal staining*: Brains were removed from adult mice and fixed similarly to embryos. Fixed brains were washed, and 100-μm sections prepared using a vibratome (Leica). Sections were mounted onto glass slides, stained, washed and dehydrated similarly to embryonic brains. Sections were mounted with cover glass using Mowiol (Calbiochem, 475,904) medium and stored at 4°C.

### Golgi-cox silver staining and neuronal reconstruction

*Golgi staining*: Neurons in intact brains were stained using the Rapid GolgiStain Kit [FD Neurotechnologies, PK401]. Staining was carried out as per manufacturer’s protocols, briefly; removed brains were impregnated with solution in darkness, stained for 2 weeks, and sectioned using a vibratome (Leica) at a thickness of 100 μm. Slices were transferred to gelatin coated slides (0.3% gelatin [Sigma-Aldrich, G9391], 0.05% chromium potassium sulfate [Acros Organics, 222,521,000] in H_2_O) and dried. Dried slices were stained using kit working solutions, dehydrated using increasing concentrations of EtOH and cleared using xylene (Fisher scientific, 10,385,910) prior to mounting with per mount (Fischer scientific, 15,832,544) and covering with coverglass.

*Neuronal reconstruction*: Prepared sections were imaged, traced and analyzed using Neurolucida software (MBF Bioscience). Neurons were traced live in X, Y and Z dimensions using and reconstructed and analyzed using inbuilt sholl-analysis tools within Neurolucida.

### Hippocampal and cortical neuronal culture and transient transfection

*Hippocampal/Cortical neuronal cultures*: Hippocampal/Cortical cultures from crosses of heterozygous *Ap4e1* animals were prepared from embryos at E16 as described previously [76–78]. Briefly, hippocampi and cortices were dissected in ice-cold HBSS [Gibco, 14,180–046] supplemented with 10 mM HEPES, pH 7.5 and incubated in 0.25% trypsin (Sigma-Aldrich, T4799) for 15 min prior to trituration. Dissociated neurons were seeded onto poly-L-lysine (Sigma-Aldrich, P2636; 0.5 mg/ml in 0.1 M borate buffer, pH 8)-coated coverslips at a density of 30–50,000/cm^2^ in attachment medium (10% horse serum [Gibco, 26,050,088], 10 mM sodium pyruvate [Gibco, 11,360,070], 0.6% glucose in MEM [Gibco, 31,095,029]). Attachment media was replaced the next day with Maintenance medium (2% B27 [Gibco, 17,504–044], 2 mM glutamax [Gibco, 35,050,061], 100 μg/ml Penicillin/Streptomycin [Gibco, 15,140–163] in Neurobasal [Gibco, 21,103,049]). Fifty percent of the maintenance medium was replaced every 4 days after the first week in culture to maintain cell health.

*Transient transfection*: Neurons were transfected using lipofectamine 2000 (Invitrogen, 1,168,019) according to manufacturer’s protocols, at an empirically determined ratio of lipofectamine to DNA per construct used (GFP 0.25 μg, RFP-LC3 0.25 μg, HA-ATG9A 1 μg and *Ap4e1* constructs 1 μg per 2 coverslips, 1 μl lipofectamine per coverslip). Neurons were left to express constructs for 2–3 days prior to further experimentation.

### Immunocytochemistry, immunohistochemistry and fluorescent dyes

*Immunocytochemistry (ICC*): Hippocampal cultures on coverslips were fixed prior to staining with 4% PFA with 4% sucrose (Sigma-Aldrich, S9378) in PBS for 7 min at RT. Post-fixation coverslips were washed in PBS and permeabilized for 10 min in blocking solution (1% BSA, 10% horse serum, 0.1% Triton X-100 in PBS). Primary antibodies were diluted in blocking solution at empirically determined dilutions and applied for 1 h at RT in a dark humidified chamber. Coverslips were washed in PBS and fluorescent-conjugated secondary antibodies as listed above were used at a concentration of 1:1000 and applied for 1 h at RT in a humidified chamber. Coverslips were mounted in ProLong Gold mounting medium (Invitrogen, P36930) and allowed to dry overnight at RT prior to imaging.

*Immunohistochemistry (IHC*): Brains were removed from animals and fixed by immersion in 4% PFA for 24 h at 4°C, cryoprotected in 30% Sucrose-PBS for 24 h and frozen and stored at −80°C. Frozen brains were embedded into OCT compound (Tissue-Tek, 4583) and serially cryosectioned into 30-μm sections in a Bright OTF-AS Cryostat (Bright Instruments) and stored at −20°C prior to staining in cryoprotective solution (30% glycerol, 30% PEG in PBS). IHC staining was performed with free-floating sections at RT with gentle agitation. Sections were washed and permeabilized in PBS-Tx (0.5% Triton X-100 in PBS) for 30 min prior to blocking in IHC blocking solution (3% BSA, 10% FBS, 0.2 M glycine [Fisher scientific, 10,080,160] in PBS-Tx) for 3 h. A second block was applied for 3 h as prior but with the addition of goat anti-mouse Fab-fragment (Jackson Immunoresearch, 115–007-003) at 50 μg/ml to reduce endogenous background when using antibodies raised in mouse. Sections were washed for 30 min and primary antibodies applied at concentrations as listed above in IHC blocking solution for 4 h. Sections were washed for 30 min and fluorescent antibodies applied for 4 h prior to a final wash and mounting onto glass slides with Mowiol medium. Slides were allowed to dry at RT overnight prior to imaging.

*Fluorescent dyes*: Free-floating sections as prepared for IHC were washed in PBS-Tx, and incubated at RT with gentle agitation for 4 h with FluoroMyelin Green (Invitrogen, F34651; 1:400) in blocking solution. Sections were washed for 3 × 30 min prior to mounting onto glass slides with Mowiol. Slides were allowed to dry at RT overnight prior to imaging.

### Autophagic flux assays

Cortical neurons were cultured onto 6-well dishes at a density of 80,000 per well and maintained in neuronal maintenance media as described. At DIV-8, cells were treated with 100 nM bafilomycin A_1_ (VWR, 196,000), in conditioned maintenance media, or additionally with 250 nM rapamycin (Cayman Biotech, 53,123–88-9) for durations as indicated. EBSS conditions were first washed once with EBSS (to remove excess serum form maintenance media) followed by addition of EBSS + bafilomycin A_1_. After treatment, cells were washed once in EBSS and collected directly by addition of sample buffer. Samples were sonicated, boiled and subjected to SDS-PAGE and western blotting as described.

### Correlative light and electron microscopy

Cells were cultured on gridded coverslip-bottomed dishes (MatTek, P35G-1.5–14-CGRD) to facilitate correlation between light and electron microscopy and processed for the latter as described in [79]. Brightfield images of regions of interest were acquired using an Olympus BX50WI upright microscope and TEM images acquired using an FEI tecnai G2 Spirit transmission electron microscope and an Olympus SIS Morada CCD camera.

### Live imaging, autophagosome motility analysis and long-term live imaging

*Live Imaging of autophagosome maturation*: For autophagosome maturation and motility experiments, cultured hippocampal neurons were transfected at DIV-4 with RFP-LC3 as described, to be imaged at DIV 6–7. Imaging was carried out under perfusion with ACSF (124 mM NaCl_2_, 2.5 mM CaCl_2_ [VWR, 190464k], 2.5 mM KCl [Sigma-Aldrich, P9541], 1 mM MgCl_2_ [Melfords, M0535], 10 mM D-glucose [Fisher scientific, G/0500/53], 25 mM NaHCO_3_ [Sigma-Aldrich, S5761], 1 mM Na_2_HPO_4_, pH 7.4) at 37°C with a flow-rate of 1–2 ml/min and aerated (5% CO_2_, 95% O_2_) throughout. Growth-cones were identified and the RFP-LC3 signal in the distal most 75 μm of axon captured using a EM-CCD camera system (iXon, Andor technology) mounted to an Olympus microscope (BX60M) with a 60x objective, as described previously [75]. A mercury arc lamp with filtering provided excitation of the RFP fluorophore (Cairn Research). Images were acquired using MicroManager (Opensource, Micro-manager.org) [80] for 6 min at 1 frame every 1.5 s.

*Autophagosome motility analysis*: Movies generated from distal axons used to generate kymographs using the ‘Multiple Kymograph’ plugin. Resulting kymographs represent autophagosome motion as time on the Y axis (1.5 s/px) and distance on the X (0.1333 μm/px). Trajectories were manually tracked and analyzed using an in-house MATLAB script. Briefly, the motion of an autophagosome is possible to define precisely by the positional change from co-ordinates x^1^/y^1^ to x^2^/y^2^. Calculating all of the individual trajectory changes for an individual autophagosome’s track we were able to ascertain; velocity, proportion of time spent moving, directionality etc. Per track, portions of time spent moving at less than 0.05 μm/s were classed as stationary.

*Long-term live imaging*: Neurons were seeded onto Poly-L-Lysine coated 96-well plates (Ibidi, 89,626), transfected with CAG-GFP at DIV-3/4 as described and imaged 2 days later using an ImageXpress Micro XLS imaging system (MolecularDevices) with environmental control. Timelapse movies were taken from up to 16 different fields per well at a rate of 1 frame every 5 min for 6 h (72 frames). Eight-bit images were recorded, movies generated using a custom ImageJ plugin, and regions with growing axons cropped for analysis.

### Structured illumination imaging (SIM)

SIM was performed on a commercially developed Zeiss Elyra PS.1 inverted microscope using a Zeiss 63x oil objective lens (NA: 1.4) and pco.edge CMOS camera and ZEN Black software (Zeiss) as described previously [78,79]. Images were captured using SIM paradigms (34-μm grating, 3 rotations and 5 lateral shifts) and processed using the SIM reconstruction module within ZEN Black with default theoretical PSF and other settings. Shifts between acquired channels were corrected for using 100-nm Tetraspeck fluorescent microspheres (Invitrogen, T7279).

### Image analysis

All imaging and image analysis techniques were performed blinded. All WT and KO embryos generated per genotype were used, and cell numbers kept consistent between embryos rather than genotypes (as a result of blinding at acquisition stage). Between 3 and 6 images were taken per condition and samples sizes kept consistent across experimental techniques. All microscopic imaging unless stated otherwise was using an upright Zeiss LSM700 confocal microscope. Images were digitally captured using Zen 2010 Software (Zeiss), using oil immersion objectives: 63x; 1.4 NA, 40 × 1.3 NA and air objectives; 10 × 0.3 NA, 5 × 0.16 NA.

*Brain measurements*: Quantification of the thickness and lengths of axonal tracts was performed manually using Fiji. At least 2 brain sections per animal were analyzed and the mean measurement used as the representative value.

*Axonal Length, Branching and swellings*: GFP-filled neurons at DIV-4 were fixed and imaged using a 40x objective, and images stitched using ‘MosaicJ’ or ‘Pairwise stitching’ [81] plugins in FIJI as required. Entire lengths of axons including all branches was measured manually using FIJI, and branches quantified excluding any process below 20 μm. Axonal swellings were defined as a compartment > 2x the width of the axon shaft, and numbers counted manually. For DIV-14 swellings quantification, fields of view were captured, total axonal length and numbers of swellings present within the field captured were counted to determine swellings per 100 μm of axon.

*Dendritic morphology and complexity*: DIV-14 GFP-filled neurons were fixed and imaged, and images stitched as previously described where necessary. Dendritic morphology was reconstructed using Neuronstudio (CNIC) and inbuilt analysis tools used to ascertain total dendritic length and branches as described previously [75,82]. Sholl analysis of intersections was performed using the ‘Simple Neurite Tracer’ plugin in FIJI, with a sholl radius of 10 μm. Images were stitched where necessary.

*Nascent dendritic length and branching*: DIV-4 GFP filled neurons were reconstructed using Neuronstudio and total length and branches of nascent dendritic processes per neuron quantified using inbuilt tools.

*ICC quantification of total fluorescent signal*: For quantification of dendritic and axonal vesicle numbers, regions positive for compartment markers (MAP2 and NEFH/NF200 respectively) were outlined manually per image, and values normalized to area. Total fluorescence of ATG9A was quantified by outlining the cell soma and measuring total fluorescence using inbuilt FIJI tools. GFAP immunoreactivity was quantified using total fluorescence per hippocampal region.

*Neuronal cell number quantification*: For neuronal cell number measurements, sections stained against RBFOX3/NeuN were imaged and RBFOX3/NeuN-positive cells counted. For relative cortical neuronal density, 100-μm wide columns of the cortex were cropped and counted (2 sections per animal); for regions of the hippocampus, 30 μm regions were cropped and counted (2 sections per animal).

*Puncta size, number and association*: For quantification of sizes and numbers of puncta, DIV-8 hippocampal neurons were stained using relevant antibodies and processed and imaged as described. Puncta size and number were analyzed using in-built tools within Metamorph software (Molecular Devices). Signals for LC3 and WIPI2 were blindly thresholded over entire sample sets, puncta manually segmented where required (when overlapping or in indiscernible proximity when threshold applied) and puncta number and area quantified with an exclusion cut-off set at 0.05 μm^2^. Puncta association (WIPI2 and LC3) was quantified using inbuilt integrated co-localization tool.

### Statistical analysis

Results were analyzed using Graphpad Prism 6 (Graphpad Software Inc). Data is presented as mean ± SEM. Where normalized, values are presented relative to the average of control values unless stated otherwise. Data was tested for normality prior to statistical testing, and appropriate statistical tests used. For differences between 2 groups statistical significance was determined using unpaired two-tailed Student’s t-tests when parametric. Two groups were tested using two-tailed Mann-Whitney U tests where at least one group was non-parametric. For 3 or more groups, statistical significance was determined by two-way ANOVAs with Bonferroni post-hoc testing where data was parametric. Kruskall-Wallis H tests were used for comparison of three or more groups where at least 1 group was non-parametric. Significance is represented as; p* < 0.05, p** < 0.01 and p*** < 0.001.
