# Supplementary material for: Axonal autophagosome maturation defect through failure of ATG9A sorting underpins pathology in AP-4 deficiency syndrome
Source: Autophagy. 2019 May 29;16(3):391–407. doi: 10.1080/15548627.2019.1615302 (PMC6999640; doi:10.1080/15548627.2019.1615302)
Supplement: Supplemental Material [file kaup-16-03-1615302-s009.zip › Supplementary information/Supplementary figure captions.docx]

**Figure S1.** *ap4e1* null mice recapitulate neuroanatomical features of AP-4 deficiency. (**A**) Representative genotyping PCR of litter used for E16 hippocampal neuronal culture showing *Ap4e1* ^(+/+)^ (WT), *Ap4e1* ^(-/-)^ (KO) and *Ap4e1* ^(+/-)^ (Het) embryos. Bottom panel, western blot showing loss of AP4E1 protein in KO embryos. (**B**) Embryos at E12.5 and E13.5, and (**C**) brains at E15.5 showing expression of promoter driven *Ap4e1* tm1b cassette throughout all visible tissues as revealed by beta-galactosidase assay. (**D**) Coronal and sagittal sections of adult heterozygous brains showing expression of tm1b cassette throughout brain. HET shown due to lack of reporter cassette in WT. (**E**) Sections from 1-month-old mice stained against FluoroMyelin showing no enlargement of third and fourth ventricles. Scale bar: 100 μm. (n = 3 animals). (**F – H**) Sections from 1-month-old mice stained using FluoroMyelin showing (**F**) anterior commissure, (**G**) external capsule and (**H**) lateral optic tracts. Scale bar: 100 μm. (n = 3 animals). (**I**) Regions of cortex and quantification of cortical neuronal density in sections prepared from *Ap4e1* line at 1-month and 4-month timepoints (relates to Fig 1C) (n = 3 animals). (**J - N**) Relative neuronal cell number and astrocytic localization in CA1, CA3 and DG regions of the hippocampus at 1-month and 4-month timepoints. Scale bar: 50 μm. (**K, L**) Quantification of relative neuronal numbers. (**M, N**) Quantification of relative GFAP immunoreactivity (n = 3 animals). Quantified data expressed as mean ± SEM. Statistical analysis: Two-tailed unpaired Student’s t-test, ^*^p<0.05. CC – corpus callosum.

**Figure S2.** ATG9A handling is affected *in vivo* in *ap4e1* KO mice. (**A**) Western blot of ATG9A showing accumulation in hippocampus, cortex and cerebellar regions of KO mouse brain (n = 3 independent experiments). (**B**) Western blot of lysates prepared from brain, heart, liver and lung show accumulation of ATG9A through all investigated tissues (n = 3 independent experiments). Hip, hippocampus; Ctx, cortex; Cer, cerebellum.

**Figure S3.** ATG9A accumulates within the TGN in *ap4e1* KO neurons. (**A**) DIV-8 cultures stained against neuronal (MAP2) and astrocytic (GFAP) markers showing accumulation of ATG9A within both neuronal and astrocytic (asterisk) cell somas. Scale bar: 20 μm (n = 15/20 cells WT/KO). (**B**) WT DIV-8 cultured hippocampal neurons stained against AP4E1 and GOLGA1/Golgin-97/GOLG97. Dashed box shows magnified region and localization of AP4E1 (ε) at the TGN. Scale bars: 10 μm, 2 μm crop (n = 3 experimental repeats). (**C**) Schematic of n-terminally MYC-tagged constructs generated for this study, showing structural position of pathological premature stop mutation, V454X. Quantified data is expressed as mean ± SEM. Statistical analysis: (**A**) Two-tailed unpaired Student’s t-test, ^***^p<0.001.

**Figure S4.** Defective autophagosome maturation in *ap4e1* KO neurons. (**A**) Western blot of endogenous WIPI2 in KO hippocampus at 1 month. (**B**) Quantification of relative protein level of WIPI2 (n = 3 animals). (**C**) Western blot of ubiquitin in KO hippocampus at 1 month, showing no increase in ubiquitinated proteins in KO brains. (**D**) Quantification of relative ubiquitin protein (n = 3 animals). (**E**) DIV-8 cultured hippocampal neurons stained against endogenous WIPI2, LC3 and NEFH/NF200. Scale bar: 10 μm. (**F**) Quantification of relative numbers of WIPI2 and LC3 puncta per neuronal soma, (**G**) area of WIPI2 and LC3 puncta and (H) percentage of LC3 associated with WIPI2 (n = 31/24 neurons WT/KO). Quantified data is expressed as mean ± SEM. Statistical analysis: (**B and D**) Two-tailed unpaired Student’s t-test, (F - H) Mann-Whitney U test, ^*^p<0.05.

**Figure S5.** Axon specific defects in *ap4e1* KO neurons. (**A**) Representative reconstructions of dendritic arbors of Golgi silver stained CA1 hippocampal neurons prepared from *ap4e1* line at 4 months of age. Scale bar: 50 μm**.** (**B**) Analysis of dendritic complexity of apical dendrites (**C**) and basal dendrites. (**D**) Quantification of total dendritic length of apical and basal dendrites and (**E**) total dendritic branching of apical and basal dendrites (n = 9 neurons). (**F**) Axonal field of cultured GFP-filled DIV-14 hippocampal neurons stained against GFP showing axonal morphology. Scale bar: 20 μm. (**G**) Quantification of axonal swellings per 100 μm (n = 3/5 embryos WT/KO). (**H**) DIV-2 KO neurons showing increased axonal ATG9A upon exogenous expression of HA-ATG9A, scale bar: 10 μm (n = 14/14 neurons WT/KO). Quantified data is expressed as mean ± SEM. Statistical analysis: (**B and C**) Two-way ANOVA with Bonferroni post-hoc test, (D, E and G) two-tailed unpaired Student’s t-test, (I) Two-tailed Mann-Whitney U test, ^***^ p<0.001.

**Figure S6.** Distal generation of axonal swelling comprised of ER accumulations in AP-4 deficiency. (**A**) Long-term imaging of DIV-6 hippocampal neurons, taken at 1 frame per 5 mins. Swellings formed in WT axons disassemble rapidly, whereas KO swellings persist at their site of deposition for dramatically longer durations (formation indicated by red arrow, disassembly by blue arrow; Refer to movies S5 to S8). Scale bar: 5 μm. (**B**) Cultured DIV-4 hippocampal neurons were identified by DIC (B) for CLEM, showing correlation of axonal region, scale bars: 20 μm, 10 μm. Individual regions (growth cones) (**C**) and (**D**) shown at higher magnification, scale bar: 2 μm. Cropped regions of **C** indicated by blue and green dashed boxes, (**Ci**) and (**Cii**) respectively, showing extension of ER into the growth cone. (**E**) Swelling (**Ei**) and (**Eii**), showing ER within initial regions of growth cone, not that these growth cone expansions of the axon are largely devoid of ER, refer to Figure 6C-E for KO example, scale bar: 0.5 μm. (n = 3 experimental repeats).

**Movies S1 and S2.** Defective distal axonal autophagosome maturation in *Ap4e1* WT and KO neurons. **(S1 and S2)** Movies showing DIV 6-7 cultured hippocampal neurons transfected with RFP-LC3, pseudo-colored with range indicating LUT. Rightward motion is retrograde towards the soma, leftward is anterograde towards the growth cone. S1 is representative movie from WT and S2 is representative movie from KO. (1 frame/1.5 s for 240 frames, playback at 20 frames / second). Scale bar: 10 μm.

**Movies S3, S4, S5- S8.** Axonal swellings are *de novo* generated distally.

DIV-6 hippocampal neurons transfected with GFP enabled tracking of axonal extension live over durations of 6 h. Axonal swellings in KO neurons are formed distally, in close proximity to the growth cone, and disassemble over the range of several hours.

S3 - S4. (**S3 and S4**) Representative axons shown over 6 h. S3 WT axon, S4 KO (72 frames, 1 frame / 5 min, 12 frames / s playback). (**S5-S8**) Regions from axons S3 and S4 shown cropped, with slower playback revealing transient axonal swelling formation in WT axons, which persist for durations in the region of 15 min. KO swellings persist for hours (**S5 and S6** WT axons; **S7 and S8** KO axons; 10 frames, 1 frame/5 mins, 3 frames/s playback).

**Table S1.** Quantification of LC3-II and SQSTM1/p62 accumulation in *ap4e1* KO neurons.
