# Supplementary figures and images for: Axonal autophagosome maturation defect through failure of ATG9A sorting underpins pathology in AP-4 deficiency syndrome
Source: Autophagy. 2019 May 29;16(3):391–407. doi: 10.1080/15548627.2019.1615302 (PMC6999640; doi:10.1080/15548627.2019.1615302)

Fig S1

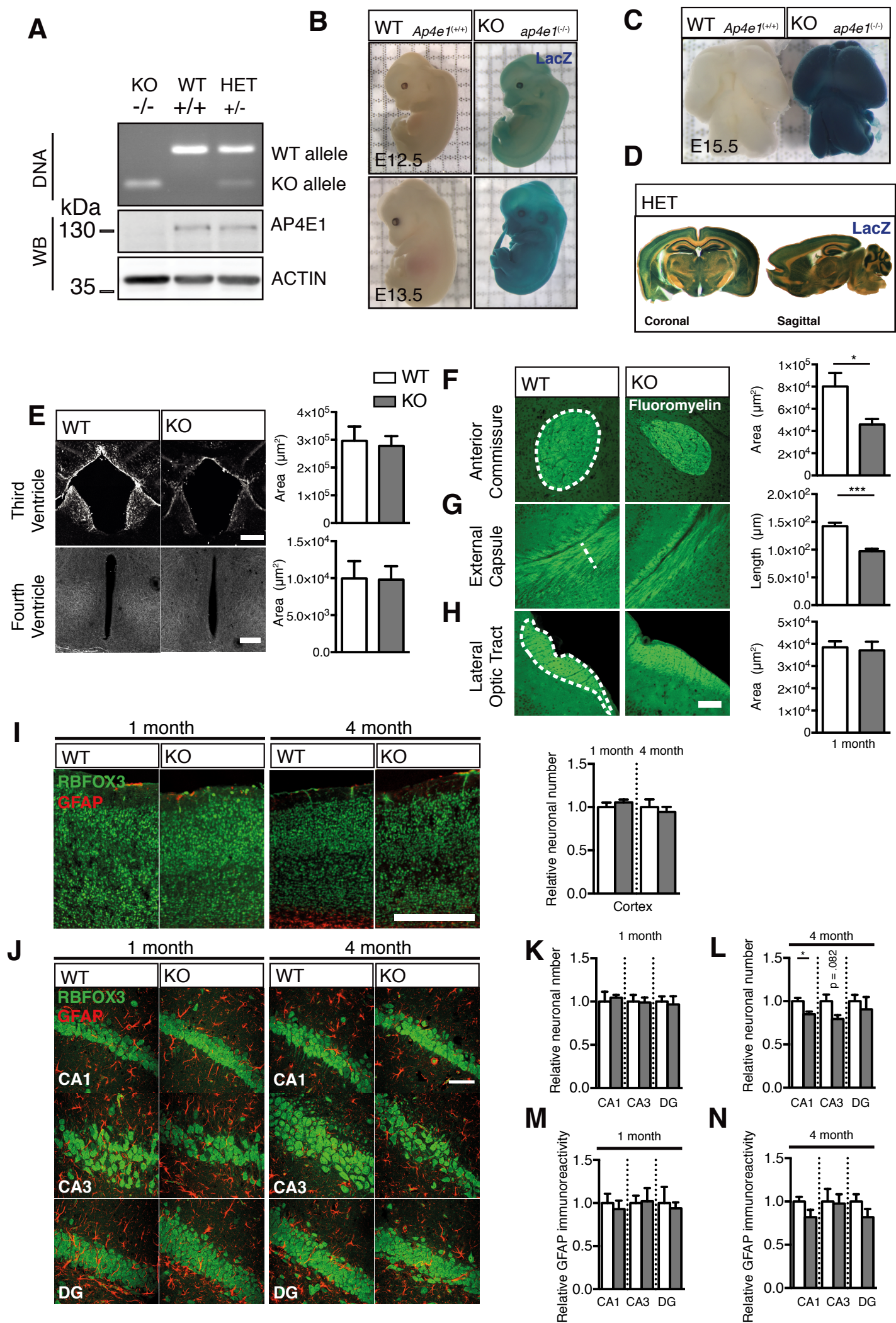

Fig S2

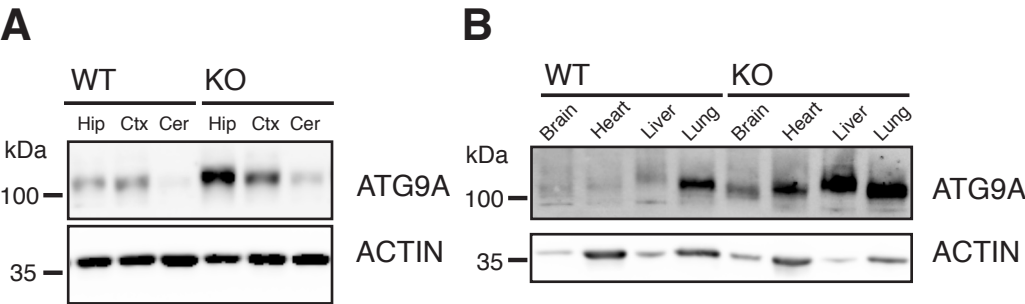

Fig S3

A

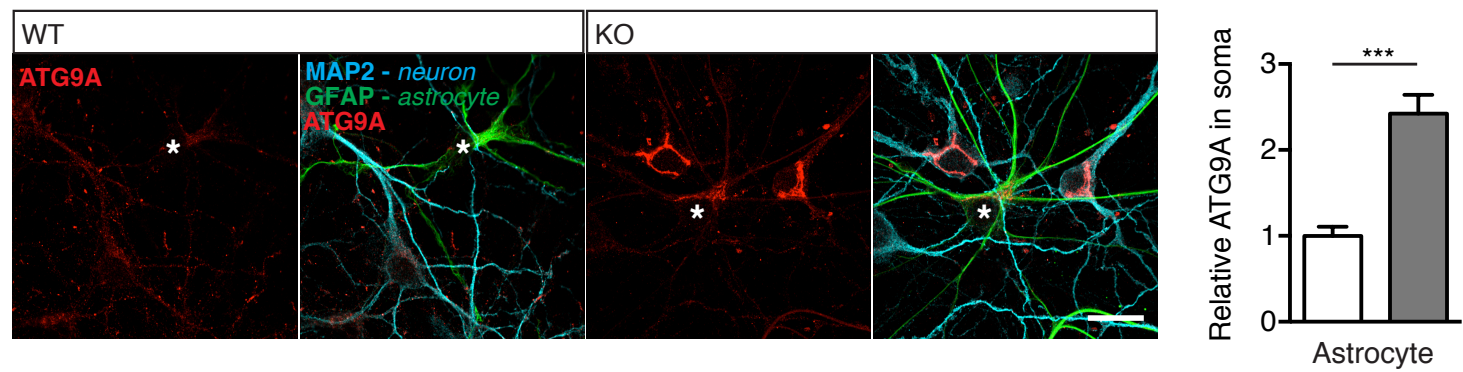

B

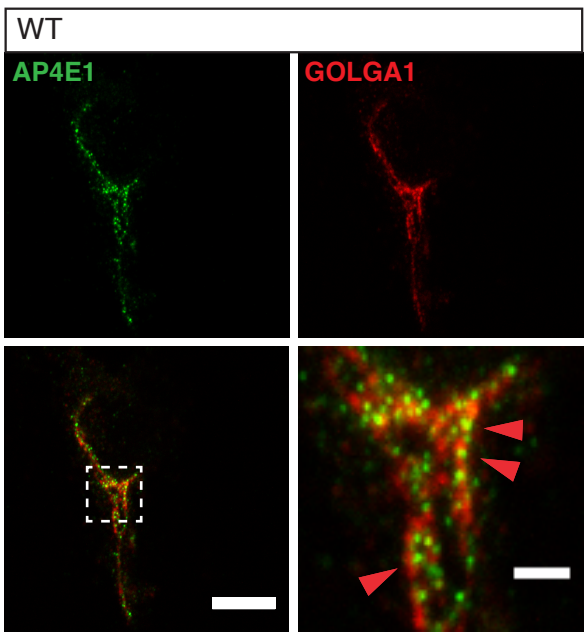

C

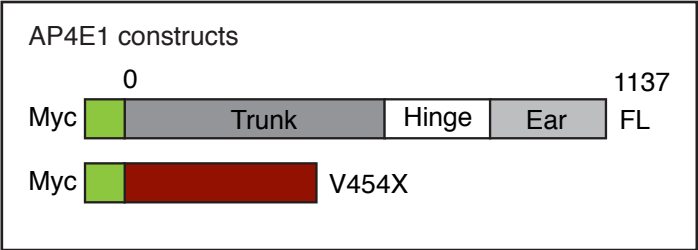

Fig S4

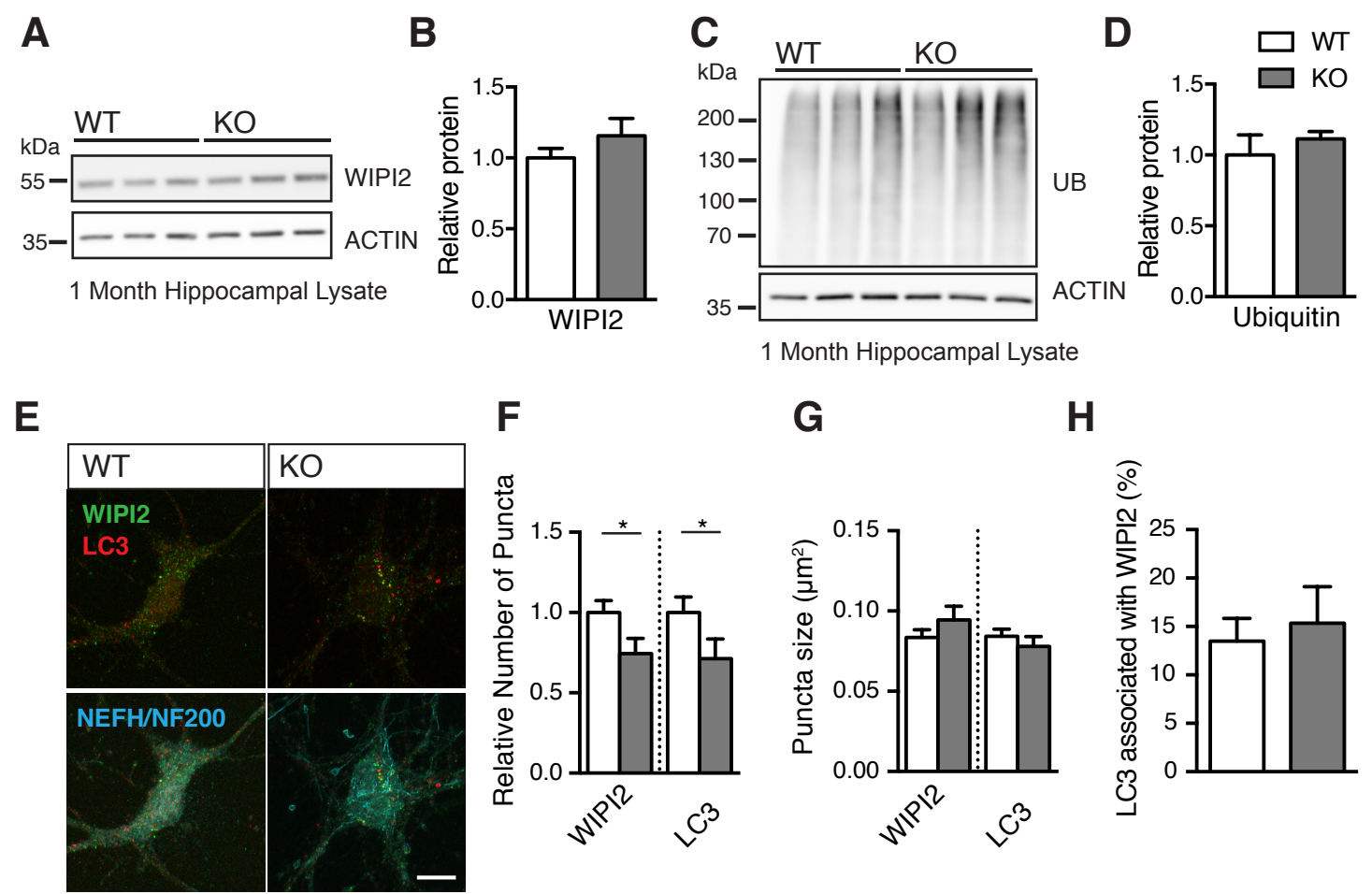

**Fig S5**

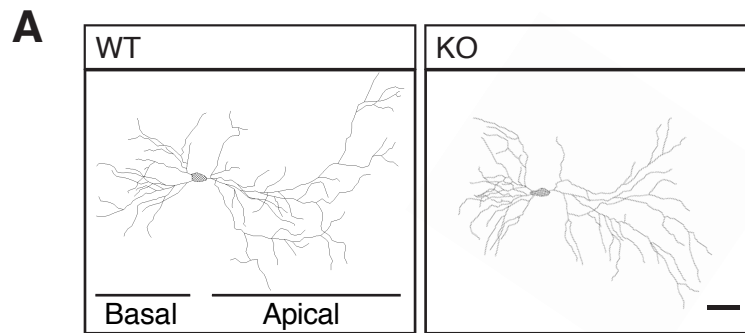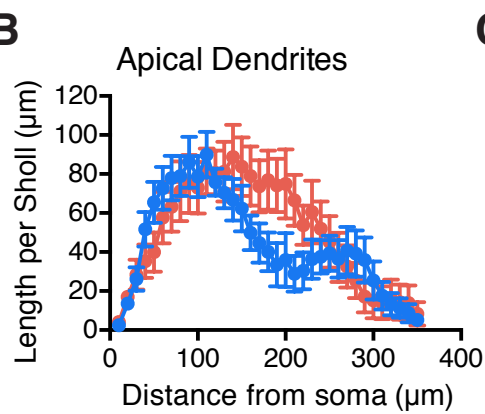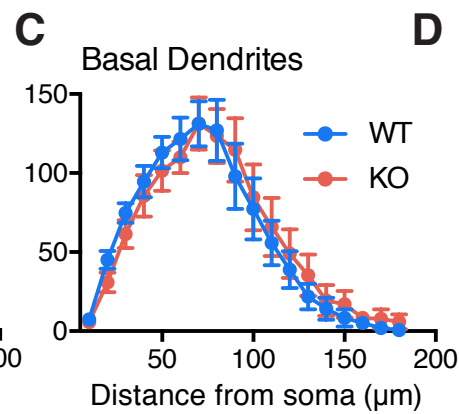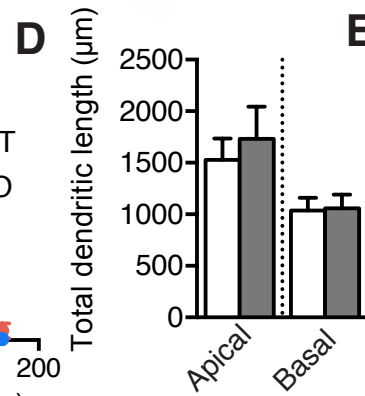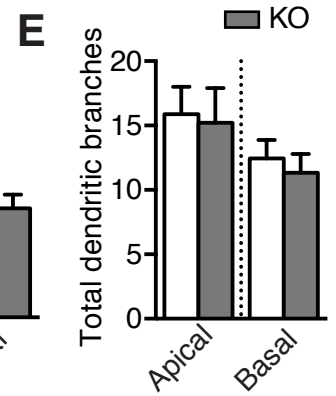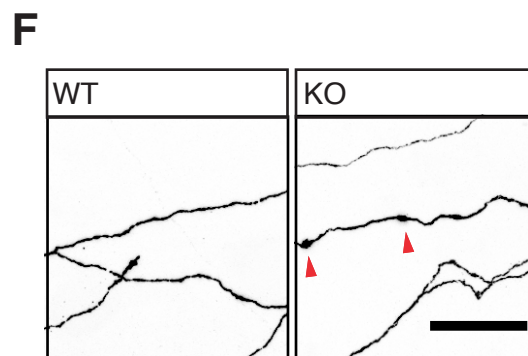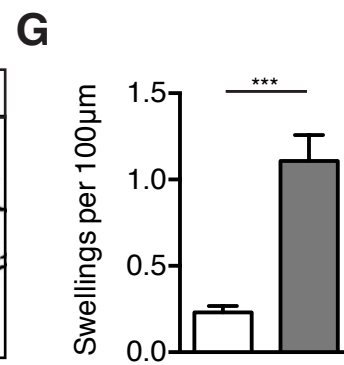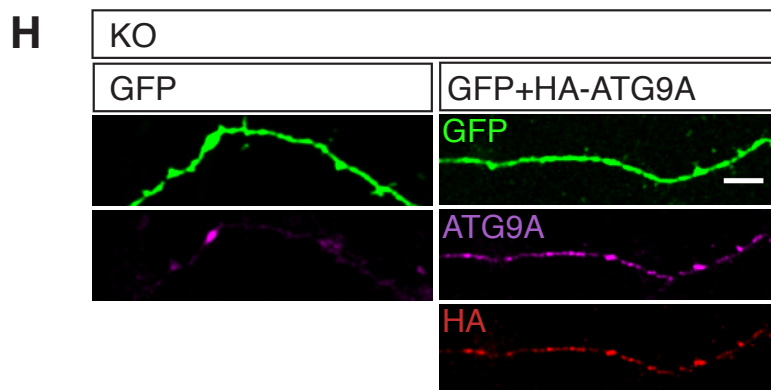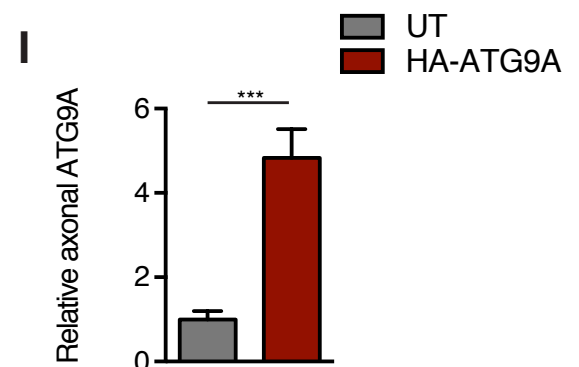

**Fig S6**

**A**

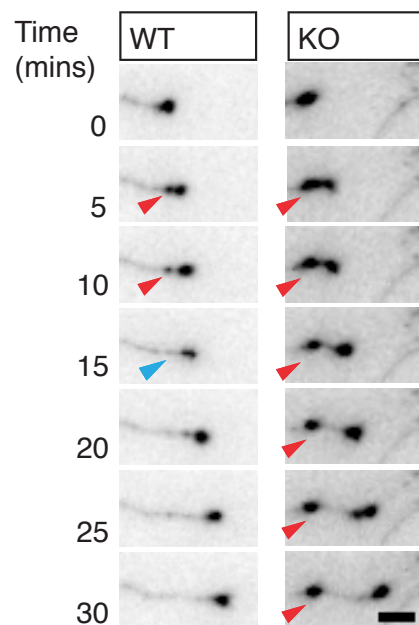

**B**

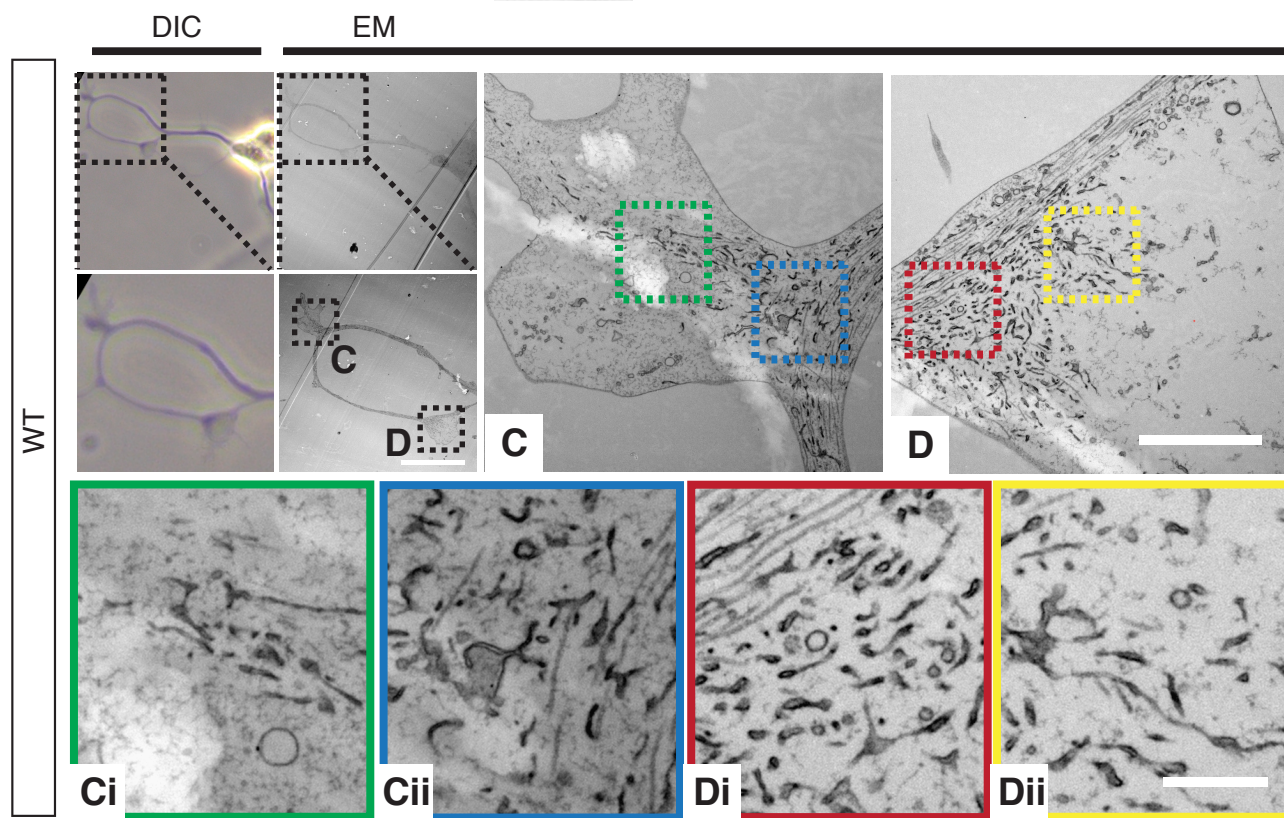

**E**

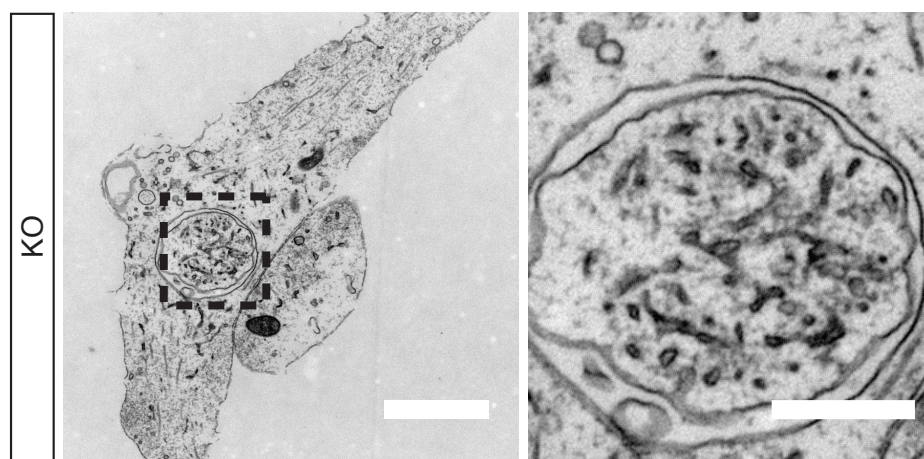

Supplement: Supplemental Material [file kaup-16-03-1615302-s009.zip › Supplementary information/Figs_Supp_R4.pdf]
